# Overall survival with immune checkpoint inhibitors across treatment settings and clinical and PD-L1-defined subgroups in advanced esophageal squamous cell carcinoma: a systematic review and meta-analysis of randomized trials

**DOI:** 10.3389/fimmu.2026.1901673

**Published:** 2026-09-03

**Authors:** Bing Wang, Xiaoyu Han, Zengli Shen

**Affiliations:** Pathology Department, Tongde Hospital of Zhejiang Province, Hangzhou, Zhejiang, China

**Keywords:** esophageal squamous cell carcinoma, immune checkpoint inhibitor, meta-analysis, overall survival, PD-L1, ratio of hazard ratios, treatment-effect modification

## Abstract

**Introduction:**

To quantify overall-survival effects of immune-checkpoint-inhibitor (ICI)-containing regimens by treatment line and to evaluate whether clinical characteristics or PD-L1 definitions modify relative treatment benefit in advanced esophageal squamous cell carcinoma (ESCC).

**Methods:**

PubMed, Embase, the Cochrane Central Register of Controlled Trials, and Web of Science were searched from inception through 1 August 2026. Two reviewers independently screened records, assessed full texts, extracted data, and evaluated risk of bias. Reports were linked to their parent randomized trial family. First-line single-checkpoint blockade plus chemotherapy and later-line checkpoint monotherapy were synthesized separately using REML random-effects models and modified Hartung-Knapp inference. Treatment-effect modification was quantified using within-trial ratios of hazard ratios (RHRs). Twenty-one interactions with at least two independent trial families entered Holm correction; Bonferroni correction was used as a sensitivity analysis.

**Results:**

We identified 14 independent randomized trial families represented by 31 reports. The primary overall-survival analysis included 7 first-line trials (*N* = 4,151; pooled HR 0.70, 95% CI 0.64–0.77; P < 0.001; *I*^2^ = 0%) and 5 later-line trials (*N* = 1,970; pooled HR 0.74, 95% CI 0.64–0.85; P = 0.004; *I*^2^ = 0%). Of 30 post hoc line-specific interaction hypotheses, 28 had at least one eligible trial-family contrast: 21 met the k ≥ 2 pooling threshold and were included in multiplicity-adjusted analyses, 7 were single-trial descriptive contrasts, and 2 had no eligible estimate. None of the 21 pooled interactions remained significant after Holm correction; all Holm- and Bonferroni-adjusted P values were 1.000. The smallest unadjusted P value was 0.092 for first-line CPS ≥ 1 versus < 1.

**Discussion:**

ICI-containing regimens improved overall survival in both first- and later-line settings, but aggregate within-trial interaction analyses did not identify a reliable clinical or PD-L1-defined treatment-effect modifier. Sparse subgroup reporting and the predominantly Asian evidence base limit the precision and generalizability of these findings.

**Systematic Review Registration:**

https://www.crd.york.ac.uk/PROSPERO/, identifier CRD42024551725.

## Graphical Abstract

**Figure:**
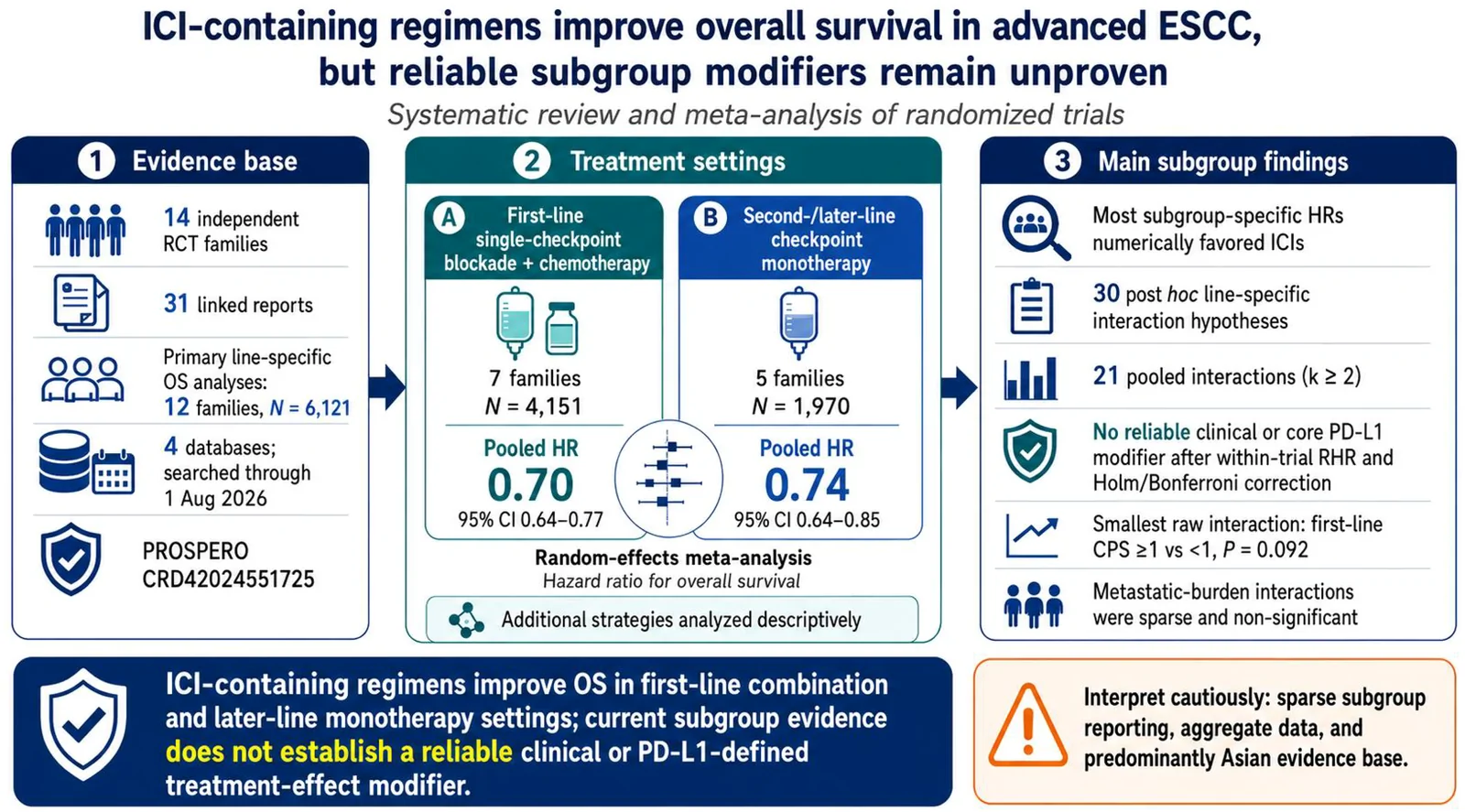
Across 12 randomized trial families involving 6,121 patients, ICI-containing regimens improved overall survival in first-line combination therapy and later-line monotherapy. None of the 21 pooled clinical or PD-L1 interaction tests remained significant after Holm correction. Subgroup findings should be interpreted cautiously because reporting was sparse and the evidence base was predominantly Asian.

## 1 Introduction

Esophageal cancer remains a major cause of cancer mortality, and esophageal squamous cell carcinoma (ESCC) accounts for most cases in high-incidence regions (1, 2). Patients with unresectable locally advanced, recurrent, or metastatic disease historically had limited survival with cytotoxic chemotherapy (3, 4). Immune checkpoint blockade targeting programmed death (PD-1) or programmed death ligand 1 (PD-L1) has changed systemic therapy in both first-line combination and later-line monotherapy settings (5–7).

Several meta-analyses have confirmed an overall survival benefit and explored clinical or PD-L1-defined subgroups (8–11). However, earlier syntheses variably combined treatment lines or strategies, counted publications rather than randomized patient cohorts, collapsed nonequivalent PD-L1 scoring systems, inferred interaction from significance within separate subgroups, or did not control for family-wise multiplicity. These choices can create apparent subgroup differences without evidence of true treatment-effect modification.

Compared with previous reviews, the search through 1 August 2026 incorporated additional randomized trial families and long-term, regional, correction, and biomarker reports. The expanded search allowed treatment-line- and scoring-system-specific within-trial ratio-of-hazard-ratio (RHR) analyses, prevented duplicate use of patients across trial updates, and supported family-wise multiplicity control (**Supplementary Table 3**). We therefore synthesized evidence at the randomized trial-family level, separated first-line from later-line therapeutic domains, preserved PD-L1 assay and scoring definitions, and quantified effect modification using within-trial RHRs. The objective was to distinguish descriptive subgroup-specific effects from statistically supported predictive modification while accounting for sparse evidence and multiple testing.

## 2 Materials and methods

### 2.1 Protocol and reporting

The review was reported in accordance with PRISMA 2020 (12). The protocol record was first submitted to International Prospective Register of Systematic Reviews (PROSPERO) on 28 May 2024 and registered on 8 June 2024 (CRD42024551725). The registered record describes database searches through 5 June 2024; registration occurred after those searches. The present review searched through 1 August 2026. The individual clinical and PD-L1 subgroup interaction hypotheses, the 30 *post hoc* hypotheses, and the multiplicity adjustment across the 21 pooled comparisons were not prespecified in PROSPERO. They were developed during the *post hoc* reanalysis and are therefore exploratory. Trial-family/report linkage and treatment-line separation were also *post hoc* methodological refinements (**Supplementary Table 8**).

### 2.2 Information sources and search

**Figure 1:**
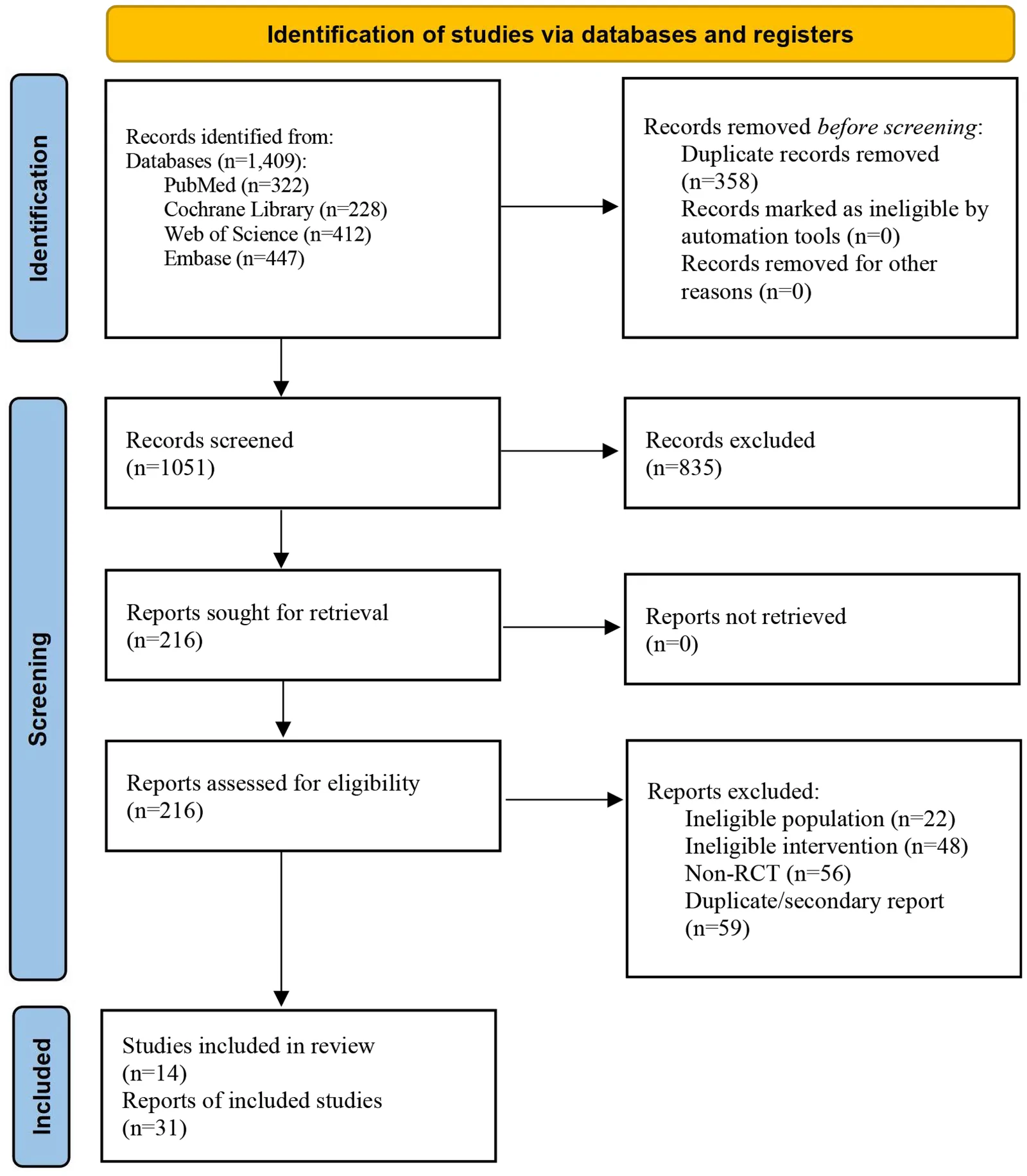
PRISMA 2020 flow diagram of study selection. PRISMA, preferred reporting items for systematic reviews and meta-analyses.

PubMed, Embase, the Cochrane Central Register of Controlled Trials, and Web of Science were searched from database inception through 1 August 2026. **Supplementary Table 2** reports the database platforms, search dates, limits, and search process. Two reviewers (B.W. and X.H.) independently screened titles and abstracts and then independently assessed potentially eligible full-text reports. Disagreements were resolved by consensus, with unresolved cases adjudicated by a third reviewer (Z.S.). Eligible primary, follow-up, biomarker, regional, and correction reports were linked to the parent randomized trial family and did not increase the trial count; the complete selection flow is shown in **Figure 1**.

### 2.3 Eligibility criteria

We included randomized controlled trials of adults with unresectable locally advanced, recurrent, or metastatic ESCC requiring systemic therapy that compared an immune checkpoint inhibitor (ICI)-containing regimen with a chemotherapy control and reported overall-survival hazard ratios (HRs) for the overall or eligible subgroup population. Mixed-histology trials were eligible only when ESCC- or squamous-cell-specific estimates were explicitly reported. Updated, biomarker, regional, and correction reports were eligible as information sources but not as additional independent trials. Nonrandomized studies, single-arm cohorts, protocols without outcome data, and reports without an eligible control contrast were excluded.

For this review, unresectable locally advanced disease meant disease not amenable to curative-intent surgery or definitive chemoradiotherapy; recurrent disease meant relapse after prior curative-intent treatment; and metastatic disease meant distant spread. These states were grouped only as an advanced systemic-therapy eligibility umbrella and were not assumed to be clinically equivalent; disease extent was retained as an explicit subgroup factor when reported.

### 2.4 Trial-family linkage and data extraction

The independent unit was the randomized trial family, not the publication. Thirty-one eligible reports were linked to 14 families (6, 7, 13–41). Each family contributed no more than one compatible estimate to a model; nested regional populations were not entered beside their global cohort, and corrections or updates did not increase the trial count. When reports differed in data cutoff, population scope, or assay definition, the report and contrast were selected according to an extraction hierarchy specified before synthesis. The report-to-trial linkage is provided in **Supplementary Table 4**.

Two reviewers (B.W. and X.H.) independently extracted trial registration, phase, treatment line and strategy, randomized and ESCC-specific sample sizes, data cutoff, follow-up, analysis population, OS HR and 95% confidence interval (CI), subgroup definition, subgroup size, PD-L1 assay, clone, scoring system, cutoff, and role in the analysis. Disagreements were resolved by consensus, with unresolved cases adjudicated by Z.S. PD-L1 combined positive score (CPS), tumor-cell/tumor proportion score (TPS), Tumor Area Positivity (TAP), vCPS, and study-specific algorithms were kept separate. ATTRACTION-3 explicitly reported no numerical body-weight/body mass index (BMI) subgroup HR; no value was imputed.

### 2.5 Risk of bias

Two reviewers (B.W. and X.H.) independently assessed risk of bias for overall survival at the randomized trial-family level using the Cochrane Risk of Bias 2 tool (RoB 2) framework (42): randomization process, deviations from intended interventions, missing outcome data, measurement of the outcome, selection of the reported result, and an overall judgment. All linked reports, corrections, protocols, statistical analysis plans, and supplementary appendices available for a family were considered, but each family appeared once in the main display. Disagreements were resolved by consensus, with unresolved judgments adjudicated by a third reviewer (Z.S.). Domain-level rationales and supporting sources are reported in **Supplementary Table 9**.

### 2.6 Statistical analysis

Overall survival was analyzed on the log-HR scale, with HR values below 1 favoring the ICI-containing regimen. First-line single PD-1/PD-L1 blockade plus chemotherapy versus chemotherapy and second- or later-line single-agent PD-1 blockade versus investigator-choice chemotherapy were synthesized separately. ASTRUM-007 enrolled only patients with PD-L1 CPS ≥ 1 and was excluded from the primary all-comer first-line OS model and primary all-comer clinical interaction family; it was retained in explicitly labeled enrichment and CPS ≥ 1-conditional sensitivity analyses. CheckMate 648 nivolumab plus ipilimumab and SKYSCRAPER-08 tiragolumab plus atezolizumab plus chemotherapy were analyzed as separate strategies. The shared CheckMate 648 chemotherapy control was not counted twice.

For each HR, log(HR) was calculated, and its standard error was derived as (log[upper 95% CI] − log[lower 95% CI])/(2 × 1.959964). Treatment-effect modification was evaluated as the within-trial RHR, defined as the HR in the numerator category divided by the HR in the complementary denominator category. The primary variance of log(RHR) was SE_1_^2^ + SE_0_^2^, assuming zero covariance; sensitivity analyses used correlation coefficients *ρ* = − 0.25, 0, and + 0.25. An RHR below 1 indicates greater relative ICI benefit in the numerator category. Statistical significance in one subgroup but not its complement was not interpreted as evidence of interaction. Category-specific subgroup HR pools were restricted to trial families reporting both complementary strata for the relevant contrast.

All pooled HR and RHR models used inverse-variance random effects with restricted maximum likelihood (REML) estimation and modified Hartung–Knapp inference with the *ad hoc* safeguard q* = max(1, *q*), so the Hartung–Knapp variance multiplier was not allowed to fall below the conventional inverse-variance value (43). Model choice was not based on *Q*, *I*^2^, or a heterogeneity *p*-value. At least two independent trial families were required for quantitative pooling. Contrasts available from one family were reported descriptively without meta-analysis; models with *k* = 2 were considered very sparse, and models with *k* = 3–4 were considered sparse. Cochran’s *Q* and its *p*-value were reported for every pooled model; prediction intervals were reported only for models with at least five independent trial families (**Supplementary Table 10**). Conventional REML-Wald, unmodified Hartung–Knapp, Paule–Mandel, and equal-effect analyses were used as sensitivity analyses. For graphical display, forest-plot markers were assigned a constant size rather than scaled by sample size, precision, or model weight; pooled estimates were displayed as diamonds, and confidence intervals extending beyond the plotting range were indicated with arrows.

Before examining the pooled interaction results, we defined 30 *post hoc* line-specific hypotheses. Twenty-eight had at least one eligible trial-family contrast: 21 met the *k* ≥ 2 pooling threshold and were included in the set adjusted for multiplicity (16 clinical all-comer models and 5 PD-L1 models with compatible scoring systems and cutoffs), seven were reported as single-trial descriptive contrasts, and two were unavailable. Holm correction across the 21 pooled interactions was primary; Bonferroni correction across the same set was a conservative sensitivity analysis. Alternative PD-L1 cutoffs and enriched definitions were analyzed separately and were not included in that adjusted set (**Supplementary Table 7**).

We also examined treatment line in exploratory univariable random-effects meta-regression using one overall OS estimate per family. This ecological comparison was not interpreted causally because line was confounded with treatment strategy. Sensitivity analyses varied the subgroup definitions, enriched populations, statistical method, covariance assumptions, and omission of individual trials. Funnel plots and regression tests for small-study effects were not performed because every factor-specific domain contained fewer than 10 independent trials. Analyses used R 4.4.0 and metafor 5.0-1 (44).

## 3 Results

### 3.1 Study selection, report linkage, and trial characteristics

The searches identified 1,409 records (PubMed, 322; Cochrane Library, 228; Web of Science, 412; Embase, 447). After 358 duplicates were removed, 1,051 records were screened, and 835 were excluded. All 216 reports sought for retrieval were obtained and assessed for eligibility. Of these, 185 were excluded: 22 for an ineligible population, 48 for an ineligible intervention, 56 as nonrandomized studies, and 59 as duplicate publications or secondary reports without additional eligible ESCC-specific OS data. Thirty-one reports representing 14 independent randomized trial families were included (**Figure 1**; **Supplementary Table 4**). The linked reports are cited collectively (6, 7, 13–41).

Nine trial families evaluated first-line therapy, and five evaluated second- or later-line therapy. Twelve families entered the primary single-checkpoint overall-OS models; ASTRUM-007 was retained as a CPS ≥ 1-enriched sensitivity trial, while CheckMate 648 nivolumab–ipilimumab and SKYSCRAPER-08 were retained as separate-strategy evidence. Trial design, eligible populations, and randomized interventions are summarized in **Table 1**. PD-L1 definitions, report linkage, analytical roles, and deduplication rules are provided in **Supplementary Tables 4**–**6**.

**Table 1:** Characteristics of the 14 randomized trial families included in the evidence synthesis.

Trial (registration)	Phase (treatment setting/region)	Eligible population and arm (*n*)	Randomized comparison	OS data cutoff	Follow-up as reported
CheckMate 648 (NCT03143153)	Phase III (first line; global)	ESCC (*n* = 970); 321/325/324	Nivolumab + chemotherapy vs. chemotherapy; nivolumab + ipilimumab separate	17 May 2022	Minimum: 28.8 months
KEYNOTE-590 (NCT03189719)	Phase III (first line; global)	Eligible ESCC (*n* = 548) (274/274); overall (*n* = 749)	Pembrolizumab + chemotherapy vs. placebo + chemotherapy	10 July 2023	5-year extended follow-up
ESCORT-1st (NCT03691090)	Phase III (first line; China)	ESCC (*n* = 596); 298/298	Camrelizumab + chemotherapy vs. placebo + chemotherapy	30 April 2022	Not reported
GEMSTONE-304 (NCT04187352)	Phase III (first line; China)	ESCC (*n* = 540); 358/182	Sugemalimab + chemotherapy vs. placebo + chemotherapy	7 October 2022	Median: 15.2 months
SKYSCRAPER-08 (NCT04540211)	Phase III (first line; Asia)	ESCC (*n* = 461); 229/232	Tiragolumab + atezolizumab + chemotherapy vs. placebo + chemotherapy	13 February 2023	Not reported
ASTRUM-007 (NCT03958890)	Phase III (first line; China)	ESCC, CPS ≥ 1 (*n* = 551); 368/183	Serplulimab + chemotherapy vs. placebo + chemotherapy	15 April 2022	Median: 14.9/15.0 months
JUPITER-06 (NCT03829969)	Phase III (first line; China)	ESCC (*n* = 514); 257/257	Toripalimab + chemotherapy vs. placebo + chemotherapy	23 February 2023	Median: 14.2 months
ORIENT-15 (NCT03748134)	Phase III (first line; multiregional)	ESCC (*n* = 659); 327/332	Sintilimab + chemotherapy vs. placebo + chemotherapy	9 April 2021	Median 16.0/16.9 months
RATIONALE-306 (NCT03783442)	Phase III (first line; global)	ESCC (*n* = 649); 326/323	Tislelizumab + chemotherapy vs. placebo + chemotherapy	28 February 2022	Median: 16.3/9.8 months
ATTRACTION-3 (NCT02569242)	Phase III (later line; global)	ESCC (*n* = 419); 210/209	Nivolumab vs. investigator-choice chemotherapy	25 May 2020	Minimum: 36.0 months
ESCORT (NCT03099382)	Phase III (later line; China)	Randomized (*n* = 457), OS FAS (*n* = 448); 228/220	Camrelizumab vs. investigator-choice chemotherapy	6 May 2019	Median: 8.3/6.2 months
ORIENT-2 (NCT03116152)	Phase II (later line; China)	ESCC (*n* = 190); 95/95	Sintilimab vs. paclitaxel/irinotecan	2 August 2019	Median: 7.2/6.2 months
KEYNOTE-181 (NCT02564263)	Phase III (later line; global)	SCC subgroup (*n* = 401); 198/203; overall (*n* = 628)	Pembrolizumab vs. investigator-choice chemotherapy	15 October 2018	Median: 7.1/6.9 months
RATIONALE-302 (NCT03430843)	Phase III (later line; global)	ESCC (*n* = 512); 256/256	Tislelizumab vs. investigator-choice chemotherapy	1 December 2020	Median: 8.5/5.8 months

Data cutoffs, median follow-up, and minimum follow-up are shown as reported and are not equivalent measures; when follow-up was unavailable, only the data cutoff is shown. Detailed doses, schedules, chemotherapy backbones, report linkage, and analytical roles are provided in **Supplementary Tables 4–6**. Mixed-histology trials contributed only explicitly ESCC/SCC-restricted estimates. ASTRUM-007 was retained only in enriched or conditional sensitivity analyses. CheckMate 648 nivolumab–ipilimumab and SKYSCRAPER-08 were analyzed as separate strategies.

### 3.2 Risk of bias

**Figure 2:**
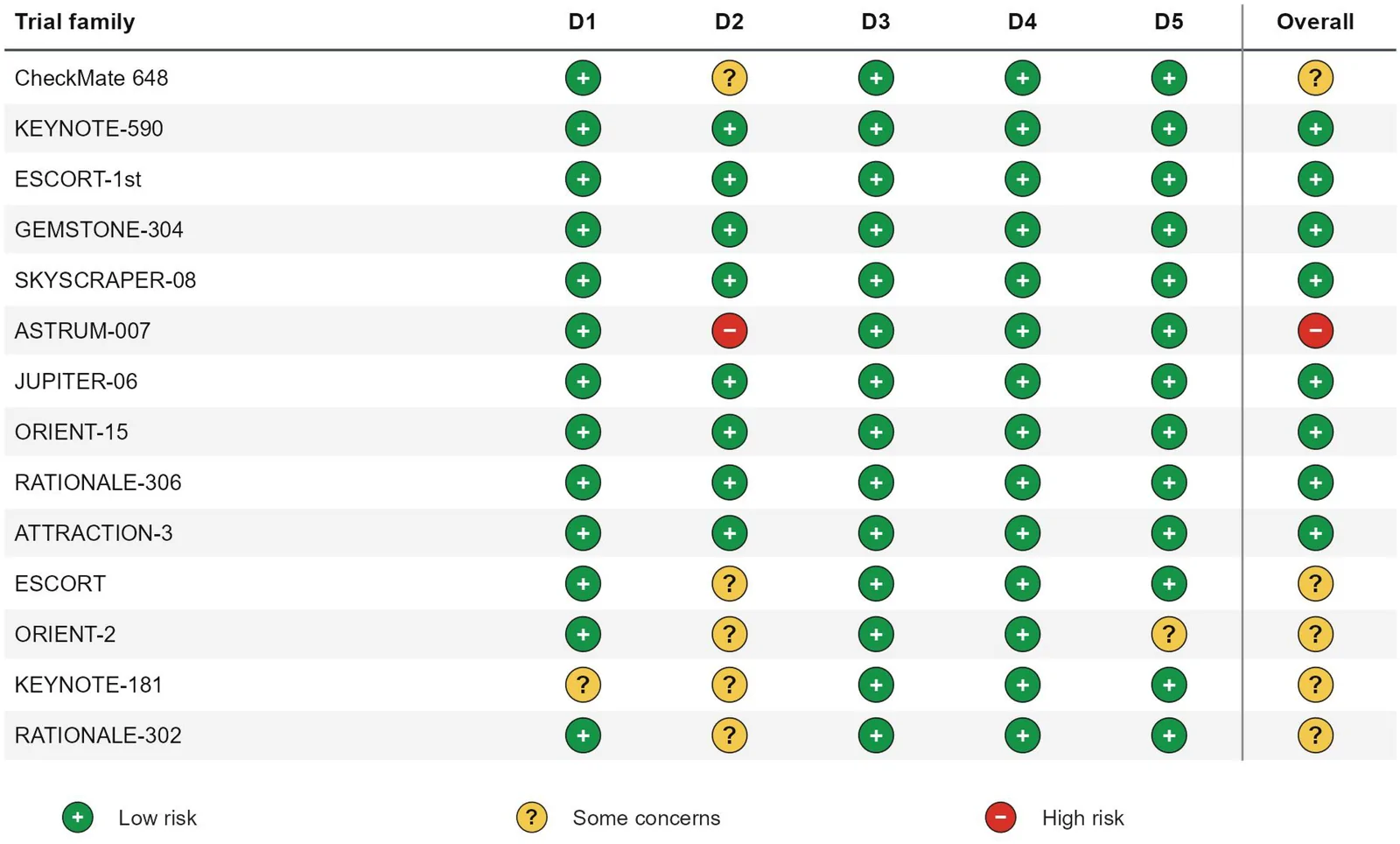
Risk-of-bias judgments for overall survival using the Cochrane RoB 2 tool. Green (+), low risk; amber ()?, some concerns; red (−), high risk. D1, bias arising from the randomization process; D2, bias due to deviations from intended interventions; D3, bias due to missing outcome data; D4, bias in measurement of the outcome; D5, bias in selection of the reported result.

Risk of bias was assessed for all 14 trial families. Overall judgments were eight low risk, five with some concerns, and one at high risk. ASTRUM-007 was judged at high risk because 15 of 183 control-assigned participants received serplulimab owing to a study-drug distribution error, an unbalanced deviation from intended intervention likely to dilute the overall-survival contrast. **Figure 2** presents family-level judgments for overall survival; the supporting report-level evidence and rationales are reported in **Supplementary Table 9**.

### 3.3 Overall survival by treatment setting

**Figure 3:**
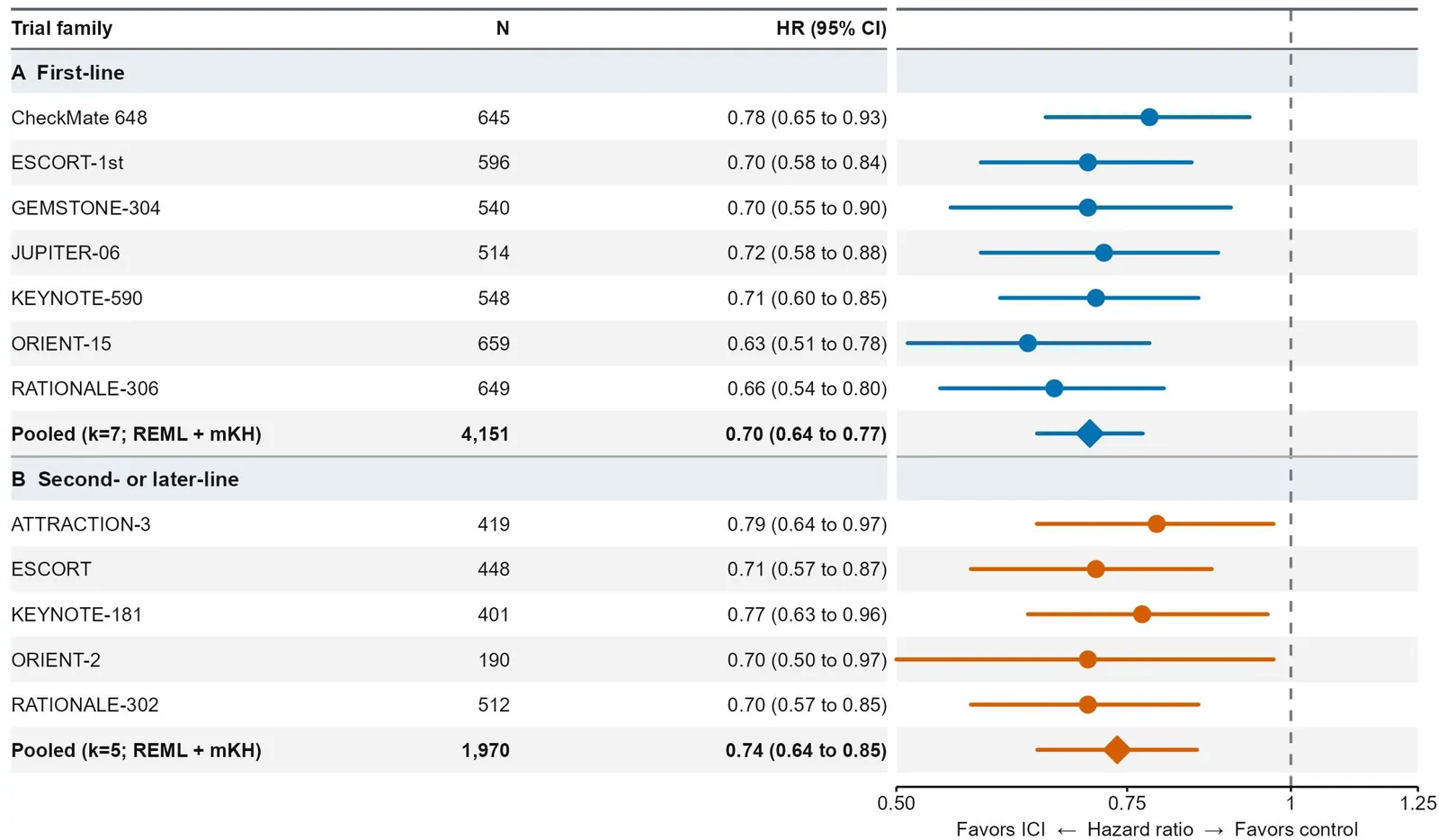
Overall-survival effects by treatment line. **(A)** First-line single PD-1/PD-L1 blockade plus chemotherapy versus chemotherapy. **(B)** Second- or later-line single-agent PD-1 blockade versus investigator-choice chemotherapy. Study markers are displayed at a constant size; diamonds denote pooled random-effects estimates. CI, confidence interval; HR, hazard ratio; ICI, immune checkpoint inhibitor; mKH, modified Hartung–Knapp; REML, restricted maximum likelihood.

In first-line single-checkpoint blockade plus chemotherapy, the pooled overall-survival hazard ratio (HR; values < 1 favor the ICI-containing regimen) was 0.70 (95% CI = 0.64–0.77; *p* < 0.001; *τ*^2^ = 0; *I*^2^ = 0%) across seven trial families and 4,151 participants. In second- or later-line checkpoint monotherapy, the pooled HR was 0.74 (95% CI = 0.64–0.85; *p* = 0.004; *τ*^2^ = 0; *I*^2^ = 0%) across five families and 1,970 participants (**Figure 3**). Although the estimated between-trial variance was zero, the small number of trials limits the ability to exclude clinically relevant heterogeneity.

### 3.4 Clinical treatment-effect modification

**Figure 4:**
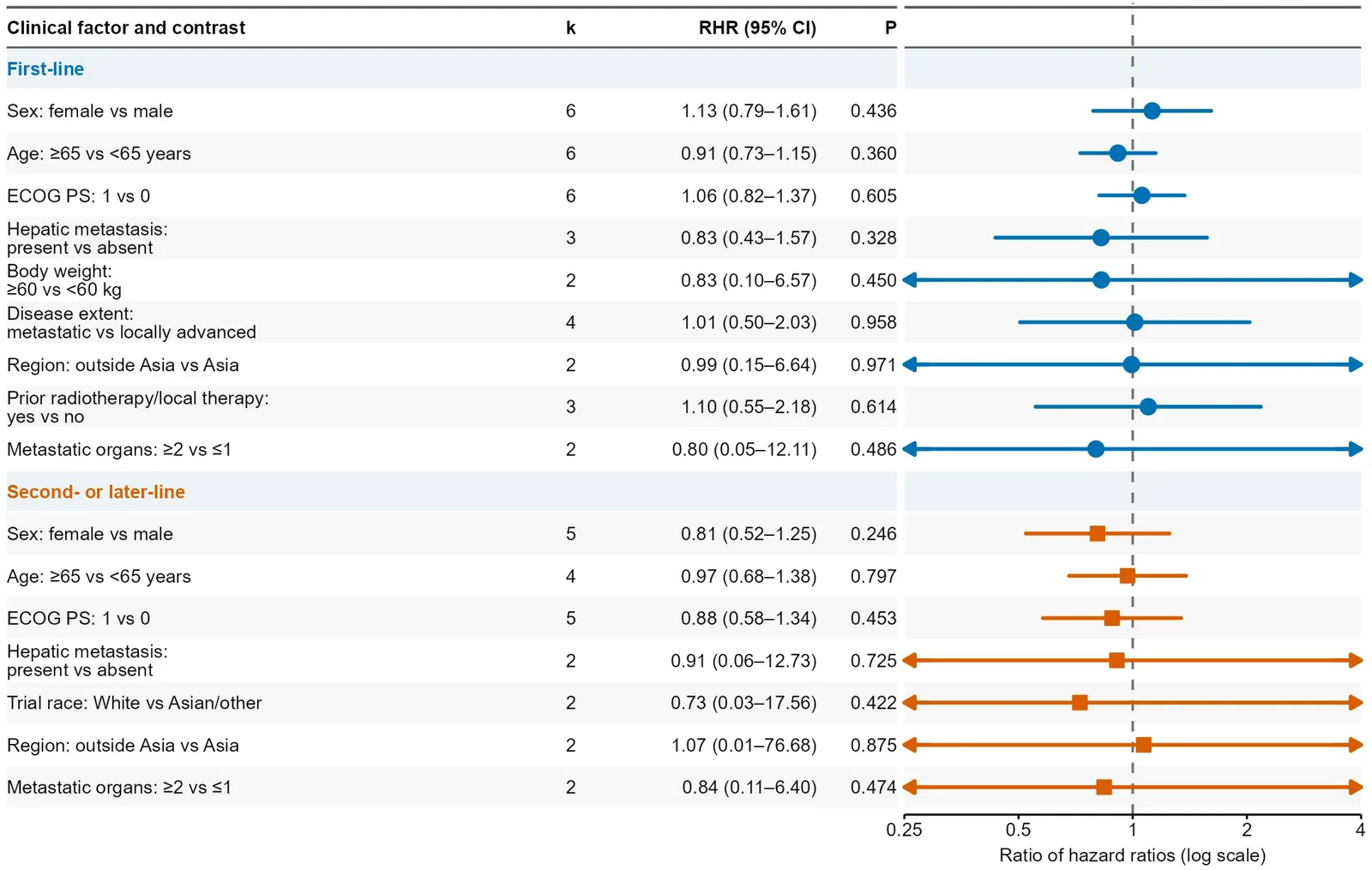
Clinical treatment-effect modification for overall survival by treatment line. Blue circles denote first-line models, and orange squares denote second- or later-line models. RHR values below 1 indicate greater relative benefit from the ICI-containing regimen in the first-named category; arrows indicate 95% CIs extending beyond the plotting range. Marker size is constant. CI, confidence interval; *k*, number of independent trial families; *P*, unadjusted interaction *p*-value; RHR, ratio of hazard ratios.

Sixteen clinical all-comer RHR models were estimable: nine first-line and seven later-line contrasts (**Figure 4**; **Supplementary Figures 1**–**11**). ASTRUM-007 was excluded from this primary set of clinical interaction analyses; its six published clinical RHRs were retained only in CPS ≥ 1-conditional sensitivity analyses. None of the 16 clinical interactions remained statistically significant after Holm correction. No evidence of treatment-effect modification by metastatic burden was observed. The first-line RHR was 0.800 (95% CI = 0.053–12.109; *k* = 2; raw *p*-value = 0.486), and the later-line RHR was 0.841 (95% CI = 0.110–6.402; *k* = 2; raw *p*-value = 0.474); both Holm-adjusted *p*-values were 1.000. Both estimates were very sparse and had extremely wide confidence intervals. The earlier nominal *p* = 0.03 finding was based on a fixed-effect comparison of subgroup-specific HRs from only ESCORT-1st and ESCORT combined across treatment lines. In the revised analysis, the nominal association was no longer present after eligible paired estimates from CheckMate 648 and ATTRACTION-3 were added and first- and later-line within-trial RHRs were analyzed separately using REML random-effects models with modified Hartung–Knapp inference. Trial-family deduplication did not remove a contributing trial family, and ASTRUM-007 did not report an eligible metastatic-burden contrast. Multiplicity adjustment was not the reason for the loss of nominal significance because both revised line-specific raw *p*-values were already nonsignificant.

### 3.5 PD-L1-defined subgroup effects and interactions

**Figure 5:**
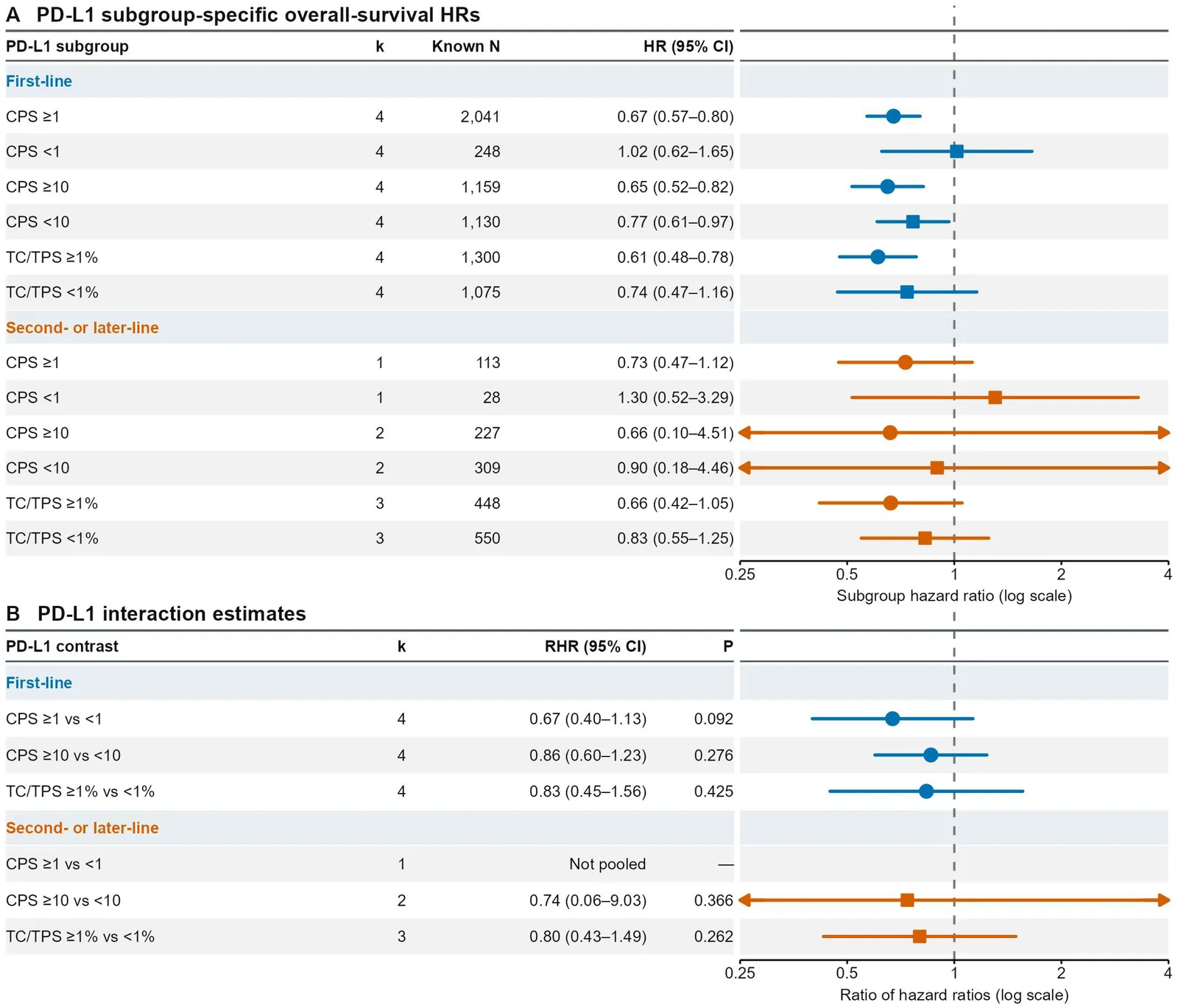
PD-L1 subgroup-specific overall-survival effects and interaction estimates. **(A)** Pooled HRs within complementary PD-L1 subgroups. **(B)** Within-trial RHRs comparing the first-named with the complementary category. In **(A)**, circles and squares distinguish the first- and second-named complementary categories; in **(B)**, blue circles and orange squares denote first- and second- or later-line models, respectively. Arrows indicate 95% CIs extending beyond the plotting range, and marker size is constant. CI, confidence interval; CPS, combined positive score; HR, hazard ratio; *k*, number of independent trial families; *N*, reported subgroup sample size; *P*, unadjusted interaction *p*-value; PD-L1, programmed death ligand 1; RHR, ratio of hazard ratios; TC, tumor cell; TPS, tumor proportion score.

Complementary PD-L1 subgroup HRs and five line-specific RHRs based on compatible biomarker definitions are shown in **Figure 5**. The minimum unadjusted interaction *p*-value was observed for first-line CPS ≥ 1 versus < 1 (RHR = 0.671, 95% CI = 0.399–1.128; *k* = 4; raw *p*-value = 0.0922), but its Holm- and Bonferroni-adjusted *p*-values were 1.000. The later-line CPS ≥ 1 versus < 1 contrast and two TAP contrasts were each available from a single trial; these estimates were reported descriptively and were neither pooled nor included in the 21 comparisons adjusted for multiplicity (**Supplementary Figure 12**). Across all 21 estimable interactions, every Holm- and Bonferroni-adjusted *p*-value was 1.000. Results based on CPS, tumor-cell/TPS, TAP, or study-specific scores were not treated as interchangeable (**Supplementary Figures 12**–**14**).

### 3.6 Descriptive clinical subgroup-specific effects

**Figure 6:**
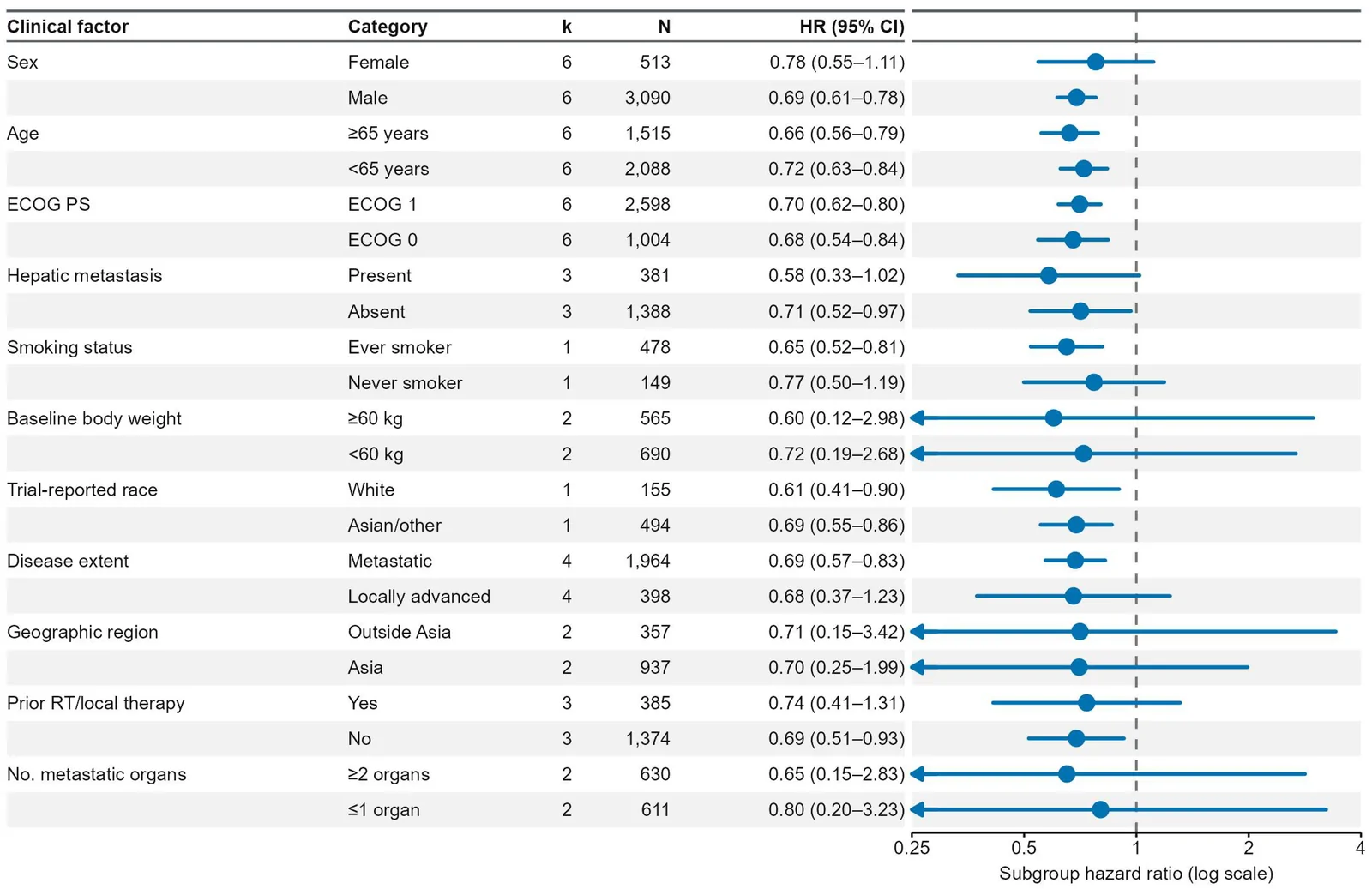
First-line clinical subgroup-specific overall-survival effects. HR values below 1 favor the ICI-containing regimen; arrows indicate 95% CIs extending beyond the plotting range. Marker size is constant. CI, confidence interval; ECOG PS, Eastern Cooperative Oncology Group performance status; HR, hazard ratio; ICI, immune checkpoint inhibitor; *k*, number of independent trial families; *N*, reported subgroup sample size; RT, radiotherapy.

**Figure 7:**
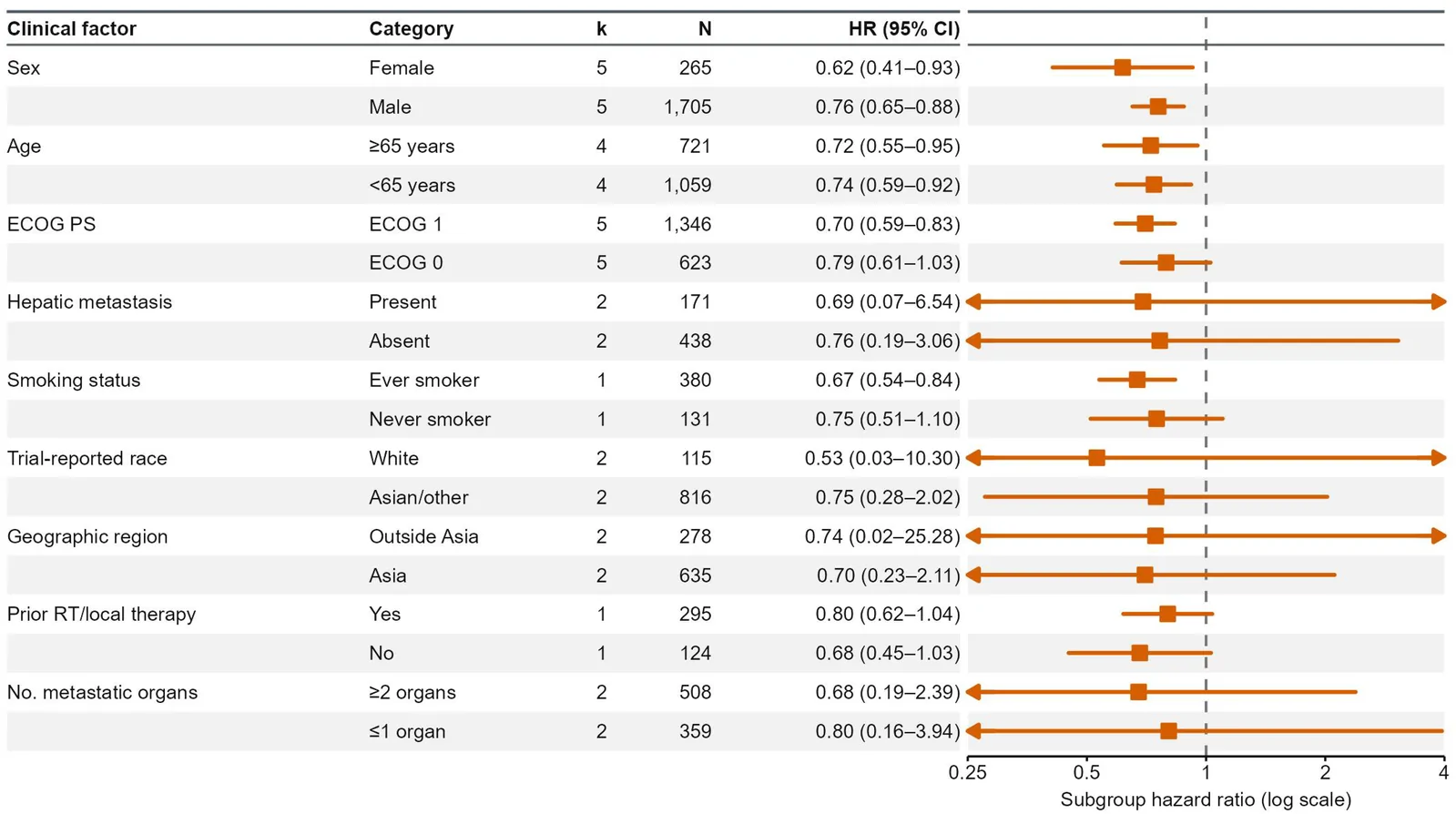
Second- or later-line clinical subgroup-specific overall-survival effects. HR values below 1 favor the ICI-containing regimen; arrows indicate 95% CIs extending beyond the plotting range. Marker size is constant. CI, confidence interval; ECOG PS, Eastern Cooperative Oncology Group performance status; HR, hazard ratio; ICI, immune checkpoint inhibitor; *k*, number of independent trial families; *N*, reported subgroup sample size; RT, radiotherapy.

First-line and later-line category-specific HRs are displayed in **Figures 6**, **7**. Most point estimates favored ICI-containing regimens, but several estimates were imprecise because few trials reported both complementary categories. These category-specific pools are descriptive and were not used to infer predictive effects from differences in within-subgroup statistical significance; formal interaction inference is provided by **Figure 4**.

### 3.7 Sensitivity and exploratory analyses

The cross-line synthesis was descriptive only and yielded an HR of 0.71 (95% CI = 0.67–0.76; 12 families; *N* = 6,121; *I*^2^ = 0%). Adding CPS ≥ 1-enriched ASTRUM-007 to the first-line sensitivity analysis produced an HR of 0.70 (95% CI = 0.64–0.76; eight families), consistent with the primary first-line estimate. Separate-strategy estimates were an HR of 0.77 (95% CI = 0.65–0.92) for CheckMate 648 nivolumab–ipilimumab and an HR of 0.70 (95% CI = 0.55–0.88) for SKYSCRAPER-08; neither was pooled into the single-checkpoint domains. The ecological later-line-versus-first-line mean-HR ratio was 1.049 (95% CI = 0.911–1.208; *p* = 0.465) and cannot be interpreted as a randomized comparison. Sensitivity analyses varying the model, covariance assumption, subgroup definition, enriched population, available estimates, and omission of individual trials did not provide multiplicity-adjusted evidence of treatment-effect modification (**Supplementary Figures 15**–**19**).

Fewer than 10 independent trial families were available in all factor-specific domains; therefore, funnel-plot asymmetry and regression tests were not undertaken. Selective nonreporting and small-study effects therefore cannot be excluded.

## 4 Discussion

ICI-containing regimens improved overall survival in both first-line combination and later-line monotherapy settings. No routine clinical or PD-L1 contrast based on compatible scoring definitions showed reliable effect modification after within-trial RHR estimation and family-wise multiplicity control. This does not prove that effects are identical for every patient; it indicates that the available aggregate subgroup evidence is insufficient to establish a differential benefit.

Prior meta-analyses evaluated overall efficacy, low PD-L1 subgroups, or clinical subgroup patterns (8–11). The updated search added randomized trial families and long-term, regional, correction, and biomarker reports not represented uniformly in those reviews. The present review links 31 reports to 14 trial families, avoids double counting across updates and regional reports, separates treatment lines and strategies, preserves PD-L1 scoring systems and cutoffs, uses formal within-trial interaction contrasts, and controls multiplicity across 21 pooled models. Accordingly, a category-specific HR pattern or nominal *p*-value was not treated as a predictive signal.

The first-line CPS ≥ 1 interaction had the smallest raw *p*-value, but its modified Hartung–Knapp CI crossed 1, and the multiplicity-adjusted result was null. The metastatic-organ estimates were based on only two trial families per treatment line and had very wide CIs. They do not provide evidence that patients with limited or oligometastatic disease derive less benefit and should not be used to select or withhold an ICI-containing regimen. Treatment decisions remain anchored to the eligible trial population, treatment setting, approved regimen, assay-specific indications, comorbidity, toxicity considerations, and patient preferences (3, 4).

PD-L1 interpretation requires particular caution. CPS incorporates tumor and immune cells, whereas tumor-cell/TPS scores incorporate tumor cells, and TAP uses a different area-based construct (6, 15, 28). Cutoffs also varied across reports and report versions or data cutoffs. Combining these definitions into a single high-versus-low biomarker would produce a clinically ambiguous estimand; we therefore retained scoring-specific models even when this reduced *k*.

The review was restricted to randomized evidence. We linked reports to trial families, used ESCC-specific estimates from mixed-histology trials, separated treatment-line and strategy domains, and applied consistent REML random-effects models, modified Hartung–Knapp inference, within-trial RHR interaction tests, multiplicity correction, and explicit reporting of sparse evidence. Linking reports in this way prevents corrections, updates, and nested regional cohorts from artificially inflating the RCT count.

Limitations include the use of published aggregate rather than individual-patient data; incomplete and potentially selective subgroup reporting; small k and low power for many interactions; unknown within-trial covariance; heterogeneous PD-L1 assays and cutoffs; *post hoc* subgroup analyses; and ecological confounding of treatment line with regimen. The evidence base was predominantly Asian, limiting inference for underrepresented non-Asian populations. Although unresectable locally advanced, recurrent, and metastatic disease were grouped under an advanced systemic-therapy eligibility umbrella, their prognosis and treatment histories differ and may contribute to residual clinical heterogeneity. *I*^2^ = 0% in the overall models does not prove clinical homogeneity. A numerical body-weight/BMI subgroup HR was unavailable for ATTRACTION-3 and was not imputed (32).

## 5 Conclusion

Across randomized ESCC trial families, ICI-containing regimens improved overall survival in first-line combination and later-line monotherapy settings. Within-trial RHR analyses, modified Hartung–Knapp inference, and Holm/Bonferroni correction did not identify a reproducible clinical or PD-L1-defined treatment-effect modifier. These exploratory aggregate subgroup findings should not be used to select or withhold an ICI-containing regimen, including for patients with limited or oligometastatic disease, particularly given sparse reporting and limited non-Asian representation.

## Data Availability

The original contributions presented in the study are included in the article/**Supplementary Material**. Further inquiries can be directed to the corresponding author.
