# Supplementary material for: Overall survival with immune checkpoint inhibitors across treatment settings and clinical and PD-L1-defined subgroups in advanced esophageal squamous cell carcinoma: a systematic review and meta-analysis of randomized trials
Source: Front Immunol. 2026 Sep 3;17:1901673. doi: 10.3389/fimmu.2026.1901673 (PMC13582564; doi:10.3389/fimmu.2026.1901673)
Supplement: Supplementary file 1 [file Table1.docx]

**Supplementary materials**

# Contents

Table S1. PRISMA 2020 checklist	2

Table S2. Database-specific search methods through 1 August 2026	3

Table S2A. Search sources, dates, and limits	3

Table S2B. PubMed search strategy	4

Table S2C. Embase.com search strategy	4

Table S2D. Cochrane Library/CENTRAL search strategy	5

Table S2E. Web of Science Core Collection search strategy	6

Table S3. Comparison with prior meta-analyses and evidence added in the present review	6

Table S4. Linkage of reports to randomized trial families	7

Table S4A. Fourteen independent randomized trial families	7

Table S4B. Thirty-one linked reports	7

Table S5. Detailed trial-family characteristics, PD-L1 definitions, and analytical roles	9

Table S5A. Trial design and randomized interventions	9

Table S5B. PD-L1 definitions and linked reports by trial family	10

Table S6. Extracted PD-L1 subgroup estimates and source locations	11

Table S7. Post hoc interaction analyses and multiplicity adjustment	16

Table S7A. Analysis status for 30 post hoc interaction hypotheses	16

Table S7B. Results of 21 pooled interaction analyses with multiplicity adjustment	17

Table S8. Departures from the registered protocol and post hoc analyses	18

Table S9. RoB 2 domain-level judgments and supporting rationale for overall survival	19

Table S10. Heterogeneity and prediction-interval statistics for primary pooled HR and RHR models	22

Figure S1. Sex subgroup-specific HRs and interaction estimates	23

Figure S2. Age subgroup-specific HRs and interaction estimates	24

Figure S3. ECOG performance-status subgroup-specific HRs and interaction estimates	25

Figure S4. Hepatic-metastasis subgroup-specific HRs and interaction estimates	26

Figure S5. Smoking-status subgroup-specific HRs and interaction estimates	27

Figure S6. Baseline-body-weight subgroup-specific HRs and interaction estimates	28

Figure S7. Trial-reported-race subgroup-specific HRs and interaction estimates	29

Figure S8. Disease-extent subgroup-specific HRs and interaction estimates	30

Figure S9. Geographic-region subgroup-specific HRs and interaction estimates	31

Figure S10. Prior-radiotherapy/definitive-therapy subgroup-specific HRs and interaction estimates	32

Figure S11. Metastatic-burden subgroup-specific HRs and interaction estimates	33

Figure S12. Core and descriptive PD-L1 interaction estimates	34

Figure S13. Secondary PD-L1 cutoff interaction analyses	35

Figure S14. Availability of PD-L1 definitions across trial families	36

Figure S15. Extended overall-survival sensitivity analyses	36

Figure S16. Sensitivity to alternative meta-analytic methods	37

Figure S17. Sensitivity to assumed within-trial correlations	38

Figure S18. Availability of eligible clinical subgroup estimates	39

Figure S19. Leave-one-out sensitivity analysis	40

## **Table S1.** PRISMA 2020 checklist

| **Section and Topic** | **Item #** | **Checklist item** | **Location where item is reported** |
| --- | --- | --- | --- |
| **TITLE** | | |  |
| Title | 1 | Identify the report as a systematic review. | Title |
| **ABSTRACT** | | |  |
| Abstract | 2 | See the PRISMA 2020 for Abstracts checklist. | Abstract |
| **INTRODUCTION** | | |  |
| Rationale | 3 | Describe the rationale for the review in the context of existing knowledge. | Introduction |
| Objectives | 4 | Provide an explicit statement of the objective(s) or question(s) the review addresses. | Introduction, final paragraph |
| **METHODS** | | |  |
| Eligibility criteria | 5 | Specify the inclusion and exclusion criteria for the review and how studies were grouped for the syntheses. | Methods 2.3 and 2.6 |
| Information sources | 6 | Specify all databases, registers, websites, organisations, reference lists and other sources searched or consulted to identify studies. Specify the date when each source was last searched or consulted. | Methods, Section 2.2; Supplementary Table S2A |
| Search strategy | 7 | Present the full search strategies for all databases, registers and websites, including any filters and limits used. | Supplementary Tables S2B–S2E |
| Selection process | 8 | Specify the methods used to decide whether a study met the inclusion criteria of the review, including how many reviewers screened each record and each report retrieved, whether they worked independently, and if applicable, details of automation tools used in the process. | Methods 2.2; Figure 1 |
| Data collection process | 9 | Specify the methods used to collect data from reports, including how many reviewers collected data from each report, whether they worked independently, any processes for obtaining or confirming data from study investigators, and if applicable, details of automation tools used in the process. | Methods, Section 2.4 |
| Data items | 10a | List and define all outcomes for which data were sought. Specify whether all results that were compatible with each outcome domain in each study were sought (e.g. for all measures, time points, analyses), and if not, the methods used to decide which results to collect. | Methods 2.3-2.6 |
|  | 10b | List and define all other variables for which data were sought (e.g. participant and intervention characteristics, funding sources). Describe any assumptions made about any missing or unclear information. | Methods, Section 2.4; Supplementary Tables S4–S6 |
| Study risk of bias assessment | 11 | Specify the methods used to assess risk of bias in the included studies, including details of the tool(s) used, how many reviewers assessed each study and whether they worked independently, and if applicable, details of automation tools used in the process. | Methods 2.5; Figure 2; Supplementary Table S9 |
| Effect measures | 12 | Specify for each outcome the effect measure(s) (e.g. risk ratio, mean difference) used in the synthesis or presentation of results. | Methods 2.6 |
| Synthesis methods | 13a | Describe the processes used to decide which studies were eligible for each synthesis (e.g. tabulating the study intervention characteristics and comparing against the planned groups for each synthesis (item #5)). | Methods 2.3 and 2.6; Supplementary Tables S4-S7 |
|  | 13b | Describe any methods required to prepare the data for presentation or synthesis, such as handling of missing summary statistics, or data conversions. | Methods 2.4 and 2.6 |
|  | 13c | Describe any methods used to tabulate or visually display results of individual studies and syntheses. | Methods, Section 2.6; main manuscript tables and figures |
|  | 13d | Describe any methods used to synthesize results and provide a rationale for the choice(s). If meta-analysis was performed, describe the model(s), method(s) to identify the presence and extent of statistical heterogeneity, and software package(s) used. | Methods 2.6; Supplementary Table S10 |
|  | 13e | Describe any methods used to explore possible causes of heterogeneity among study results (e.g. subgroup analysis, meta-regression). | Methods 2.6 |
|  | 13f | Describe any sensitivity analyses conducted to assess robustness of the synthesized results. | Methods 2.6; Supplementary Figures S15-S19 |
| Reporting bias assessment | 14 | Describe any methods used to assess risk of bias due to missing results in a synthesis (arising from reporting biases). | Methods 2.6; Results 3.7 |
| Certainty assessment | 15 | Describe any methods used to assess certainty (or confidence) in the body of evidence for an outcome. | Not assessed |
| **RESULTS** | | |  |
| Study selection | 16a | Describe the results of the search and selection process, from the number of records identified in the search to the number of studies included in the review, ideally using a flow diagram. | Results 3.1; Figure 1 |
|  | 16b | Cite studies that might appear to meet the inclusion criteria, but which were excluded, and explain why they were excluded. | Not reported; aggregate exclusion reasons are provided in Results, Section 3.1, and Figure 1 |
| Study characteristics | 17 | Cite each included study and present its characteristics. | Results 3.1; Table 1; Supplementary Tables S4-S6 |
| Risk of bias in studies | 18 | Present assessments of risk of bias for each included study. | Results 3.2; Figure 2; Supplementary Table S9 |
| Results of individual studies | 19 | For all outcomes, present, for each study: (a) summary statistics for each group (where appropriate) and (b) an effect estimate and its precision (e.g. confidence/credible interval), ideally using structured tables or plots. | Results 3.3-3.7; Figures 3-7; Supplementary Table S6 and Figures S1-S19 |
| Results of syntheses | 20a | For each synthesis, briefly summarise the characteristics and risk of bias among contributing studies. | Results, Sections 3.1–3.7; Table 1; Figure 2; Supplementary Tables S4–S10 |
|  | 20b | Present results of all statistical syntheses conducted. If meta-analysis was done, present for each the summary estimate and its precision (e.g. confidence/credible interval) and measures of statistical heterogeneity. If comparing groups, describe the direction of the effect. | Results 3.3-3.7; Figures 3-7; Supplementary Tables S7B and S10; Supplementary Figures |
|  | 20c | Present results of all investigations of possible causes of heterogeneity among study results. | Results 3.4-3.5; Figures 4-5; Supplementary Table S7; Figures S12-S14 |
|  | 20d | Present results of all sensitivity analyses conducted to assess the robustness of the synthesized results. | Results 3.7; Supplementary Figures S15-S19 |
| Reporting biases | 21 | Present assessments of risk of bias due to missing results (arising from reporting biases) for each synthesis assessed. | Methods, Section 2.6; Results, Section 3.7 |
| Certainty of evidence | 22 | Present assessments of certainty (or confidence) in the body of evidence for each outcome assessed. | Not assessed |
| **DISCUSSION** | | |  |
| Discussion | 23a | Provide a general interpretation of the results in the context of other evidence. | Discussion |
|  | 23b | Discuss any limitations of the evidence included in the review. | Discussion, limitations paragraph |
|  | 23c | Discuss any limitations of the review processes used. | Discussion, limitations paragraph |
|  | 23d | Discuss implications of the results for practice, policy, and future research. | Discussion and Conclusion |
| **OTHER INFORMATION** | | |  |
| Registration and protocol | 24a | Provide registration information for the review, including register name and registration number, or state that the review was not registered. | Methods 2.1; Supplementary Table S8 |
|  | 24b | Indicate where the review protocol can be accessed, or state that a protocol was not prepared. | Methods 2.1; Supplementary Table S8 |
|  | 24c | Describe and explain any amendments to information provided at registration or in the protocol. | Methods 2.1; Supplementary Table S8 |
| Support | 25 | Describe sources of financial or non-financial support for the review, and the role of the funders or sponsors in the review. | Declarations - Funding |
| Competing interests | 26 | Declare any competing interests of review authors. | Declarations - Competing interests |
| Availability of data, code and other materials | 27 | Report which of the following are publicly available and where they can be found: template data collection forms; data extracted from included studies; data used for all analyses; analytic code; any other materials used in the review. | Data availability statement; Supplementary Tables S4–S10 |

The checklist items are reproduced from the PRISMA 2020 statement. Locations refer to the main manuscript and supplementary material. Certainty of evidence was not assessed.

**From:** Page MJ, McKenzie JE, Bossuyt PM, Boutron I, Hoffmann TC, Mulrow CD, et al. The PRISMA 2020 statement: an updated guideline for reporting systematic reviews. BMJ 2021;372:n71. doi: 10.1136/bmj.n71. This work is licensed under CC BY 4.0. To view a copy of this license, visit https://creativecommons.org/licenses/by/4.0/

## Table S2. Database-specific search methods through 1 August 2026

### Table S2A. Search sources, dates, and limits

| **Database** | **Platform / coverage** | **Reported cutoff** | **Limits / settings** | **Final set** |
| --- | --- | --- | --- | --- |
| PubMed | NLM PubMed; inception to reported cutoff | 1 Aug 2026 | No language limit; RCT concept; animal-only exclusion | #7 |
| Embase | Embase.com; subscription source details were not documented | 1 Aug 2026 | No language/document-type limit; Cochrane 2023 RCT filter | #45 |
| CENTRAL | Cochrane Library Search Manager; database release information was not documented | 1 Aug 2026 | Trials/CENTRAL only; no added RCT/animal/language filter | #15 |
| Web of Science Core Collection | Clarivate Advanced Search; index coverage was not documented | 1 Aug 2026 | Timespan all years; no language/document-type limit | #6 |

### Table S2B. PubMed search strategy

| **Set** | **Documented syntax** |
| --- | --- |
| #1 | (("Esophageal Neoplasms"[Mesh] AND "Carcinoma, Squamous Cell"[Mesh]) OR "Esophageal Squamous Cell Carcinoma"[Mesh] OR (((esophag*[tiab] OR oesophag*[tiab]) AND (squamous[tiab] OR "squamous cell"[tiab]) AND (cancer*[tiab] OR carcinom*[tiab] OR neoplasm*[tiab])) OR ESCC[tiab])) |
| #2 | ("Immune Checkpoint Inhibitors"[Mesh] OR "Programmed Cell Death 1 Receptor"[Mesh] OR "B7-H1 Antigen"[Mesh] OR "CTLA-4 Antigen"[Mesh] OR (((immune[tiab] AND checkpoint*[tiab]) AND (inhibit*[tiab] OR block*[tiab])) OR "PD-1"[tiab] OR PD1[tiab] OR "PD-L1"[tiab] OR PDL1[tiab] OR "programmed cell death 1"[tiab] OR "programmed death 1"[tiab] OR "programmed death ligand 1"[tiab] OR "CTLA-4"[tiab] OR CTLA4[tiab] OR "cytotoxic T-lymphocyte-associated protein 4"[tiab] OR TIGIT[tiab])) |
| #3 | (nivolumab[tiab] OR pembrolizumab[tiab] OR camrelizumab[tiab] OR toripalimab[tiab] OR sintilimab[tiab] OR tislelizumab[tiab] OR serplulimab[tiab] OR sugemalimab[tiab] OR atezolizumab[tiab] OR ipilimumab[tiab] OR tiragolumab[tiab]) |
| #4 | #2 OR #3 |
| #5 | ((randomized controlled trial[pt] OR controlled clinical trial[pt] OR randomized[tiab] OR randomised[tiab] OR placebo[tiab] OR randomly[tiab] OR trial[tiab] OR groups[tiab]) NOT (animals[mh] NOT humans[mh])) |
| #6 | 1900/01/01:2026/08/01[dp] |
| #7 | #1 AND #4 AND #5 AND #6 |

### Table S2C. Embase.com search strategy

| **Set** | **Documented syntax** |
| --- | --- |
| #1 | 'randomized controlled trial'/exp |
| #2 | 'controlled clinical trial'/de |
| #3 | random*:ti,ab,tt |
| #4 | 'randomization'/de |
| #5 | 'intermethod comparison'/de |
| #6 | placebo:ti,ab,tt |
| #7 | (compare:ti,tt OR compared:ti,tt OR comparison:ti,tt) |
| #8 | ((evaluated:ab OR evaluate:ab OR evaluating:ab OR assessed:ab OR assess:ab) AND (compare:ab OR compared:ab OR comparing:ab OR comparison:ab)) |
| #9 | (open NEXT/1 label):ti,ab,tt |
| #10 | ((double OR single OR doubly OR singly) NEXT/1 (blind OR blinded OR blindly)):ti,ab,tt |
| #11 | 'double blind procedure'/de |
| #12 | (parallel NEXT/1 group*):ti,ab,tt |
| #13 | (crossover:ti,ab,tt OR 'cross over':ti,ab,tt) |
| #14 | ((assign* OR match OR matched OR allocation) NEAR/6 (alternate OR group OR groups OR intervention OR interventions OR patient OR patients OR subject OR subjects OR participant OR participants)):ti,ab,tt |
| #15 | (assigned:ti,ab,tt OR allocated:ti,ab,tt) |
| #16 | (controlled NEAR/8 (study OR design OR trial)):ti,ab,tt |
| #17 | (volunteer:ti,ab,tt OR volunteers:ti,ab,tt) |
| #18 | 'human experiment'/de |
| #19 | trial:ti,tt |
| #20 | #1 OR #2 OR #3 OR #4 OR #5 OR #6 OR #7 OR #8 OR #9 OR #10 OR #11 OR #12 OR #13 OR #14 OR #15 OR #16 OR #17 OR #18 OR #19 |
| #21 | ((((random* NEXT/1 sampl*) NEAR/8 ('cross section*' OR questionnaire* OR survey OR surveys OR database OR databases)):ti,ab,tt) NOT ('comparative study'/de OR 'controlled study'/de OR 'randomised controlled':ti,ab,tt OR 'randomized controlled':ti,ab,tt OR 'randomly assigned':ti,ab,tt)) |
| #22 | ('cross-sectional study'/de NOT ('randomized controlled trial'/exp OR 'controlled clinical study'/de OR 'controlled study'/de OR 'randomised controlled':ti,ab,tt OR 'randomized controlled':ti,ab,tt OR 'control group':ti,ab,tt OR 'control groups':ti,ab,tt)) |
| #23 | (('case control*':ti,ab,tt AND random*:ti,ab,tt) NOT ('randomised controlled':ti,ab,tt OR 'randomized controlled':ti,ab,tt)) |
| #24 | ('systematic review':ti,tt NOT (trial:ti,tt OR study:ti,tt)) |
| #25 | (nonrandom*:ti,ab,tt NOT random*:ti,ab,tt) |
| #26 | 'random field*':ti,ab,tt |
| #27 | ('random cluster' NEAR/4 sampl*):ti,ab,tt |
| #28 | ((review:ab AND review:it) NOT trial:ti,tt) |
| #29 | ('we searched':ab AND (review:ti,tt OR review:it)) |
| #30 | 'update review':ab |
| #31 | (databases NEAR/5 searched):ab |
| #32 | ((rat:ti,tt OR rats:ti,tt OR mouse:ti,tt OR mice:ti,tt OR swine:ti,tt OR porcine:ti,tt OR murine:ti,tt OR sheep:ti,tt OR lambs:ti,tt OR pigs:ti,tt OR piglets:ti,tt OR rabbit:ti,tt OR rabbits:ti,tt OR cat:ti,tt OR cats:ti,tt OR dog:ti,tt OR dogs:ti,tt OR cattle:ti,tt OR bovine:ti,tt OR monkey:ti,tt OR monkeys:ti,tt OR trout:ti,tt OR marmoset*:ti,tt) AND 'animal experiment'/de) |
| #33 | ('animal experiment'/de NOT ('human experiment'/de OR 'human'/de)) |
| #34 | #21 OR #22 OR #23 OR #24 OR #25 OR #26 OR #27 OR #28 OR #29 OR #30 OR #31 OR #32 OR #33 |
| #35 | #20 NOT #34 |
| #36 | 'esophageal squamous cell carcinoma'/exp |
| #37 | ((esophag* OR oesophag*) NEAR/3 (squamous NEAR/2 (cancer* OR carcinom* OR neoplasm*))):ti,ab,kw |
| #38 | escc:ti,ab,kw |
| #39 | #36 OR #37 OR #38 |
| #40 | 'immune checkpoint inhibitor'/exp |
| #41 | (((immune NEAR/1 checkpoint*) NEAR/3 (inhibit* OR block*)) OR 'PD-1' OR PD1 OR 'PD-L1' OR PDL1 OR 'programmed cell death 1' OR 'programmed death 1' OR 'programmed death ligand 1' OR 'CTLA-4' OR CTLA4 OR 'cytotoxic T lymphocyte associated protein 4' OR TIGIT):ti,ab,kw |
| #42 | (nivolumab OR pembrolizumab OR camrelizumab OR toripalimab OR sintilimab OR tislelizumab OR serplulimab OR sugemalimab OR atezolizumab OR ipilimumab OR tiragolumab):ti,ab,kw |
| #43 | #40 OR #41 OR #42 |
| #44 | [1900-01-01 to 2026-08-01]/pd |
| #45 | #35 AND #39 AND #43 AND #44 |

### Table S2D. Cochrane Library/CENTRAL search strategy

| **Set** | **Documented syntax** |
| --- | --- |
| #1 | MeSH descriptor: [Esophageal Neoplasms] explode all trees |
| #2 | MeSH descriptor: [Carcinoma, Squamous Cell] explode all trees |
| #3 | #1 AND #2 |
| #4 | MeSH descriptor: [Esophageal Squamous Cell Carcinoma] explode all trees |
| #5 | ((esophag* OR oesophag*) NEAR/3 (squamous NEAR/2 (cancer* OR carcinom* OR neoplasm*))):ti,ab,kw |
| #6 | ESCC:ti,ab,kw |
| #7 | #3 OR #4 OR #5 OR #6 |
| #8 | MeSH descriptor: [Immune Checkpoint Inhibitors] explode all trees |
| #9 | MeSH descriptor: [Programmed Cell Death 1 Receptor] explode all trees |
| #10 | MeSH descriptor: [B7-H1 Antigen] explode all trees |
| #11 | MeSH descriptor: [CTLA-4 Antigen] explode all trees |
| #12 | (((immune NEAR/1 checkpoint*) NEAR/3 (inhibit* OR block*)) OR "PD-1" OR PD1 OR "PD-L1" OR PDL1 OR "programmed cell death 1" OR "programmed death 1" OR "programmed death ligand 1" OR "CTLA-4" OR CTLA4 OR "cytotoxic T lymphocyte associated protein 4" OR TIGIT):ti,ab,kw |
| #13 | (nivolumab OR pembrolizumab OR camrelizumab OR toripalimab OR sintilimab OR tislelizumab OR serplulimab OR sugemalimab OR atezolizumab OR ipilimumab OR tiragolumab):ti,ab,kw |
| #14 | #8 OR #9 OR #10 OR #11 OR #12 OR #13 |
| #15 | #7 AND #14 |

### Table S2E. Web of Science Core Collection search strategy

| **Set** | **Documented syntax** |
| --- | --- |
| #1 | TS=(((esophag* OR oesophag*) NEAR/3 (squamous NEAR/2 (cancer* OR carcinom* OR neoplasm*))) OR ESCC) |
| #2 | TS=(((immune NEAR/1 checkpoint*) NEAR/3 (inhibit* OR block*)) OR "PD-1" OR PD1 OR "PD-L1" OR PDL1 OR "programmed cell death 1" OR "programmed death 1" OR "programmed death ligand 1" OR "CTLA-4" OR CTLA4 OR "cytotoxic T-lymphocyte-associated protein 4" OR TIGIT) |
| #3 | TS=(nivolumab OR pembrolizumab OR camrelizumab OR toripalimab OR sintilimab OR tislelizumab OR serplulimab OR sugemalimab OR atezolizumab OR ipilimumab OR tiragolumab) |
| #4 | #2 OR #3 |
| #5 | TS=(random* OR placebo* OR trial* OR ((single OR double OR triple) NEAR/1 (blind* OR mask*)) OR assign* OR allocat* OR group*) |
| #6 | #1 AND #4 AND #5 |

Original platform export histories were unavailable. The tables therefore present the database-specific strategy documented for the review update through 1 August 2026 rather than archived search logs.

## Table S3. Comparison with prior meta-analyses and evidence added in the present review

| **Review** | **Search end** | **Evidence described** | **Main focus/method** | **Distinguishing boundary** | **New evidence/analysis incorporated in the present review** |
| --- | --- | --- | --- | --- | --- |
| Yap et al., 2023 (PMID 36480211) | 1 Oct 2021 | 9 RCTs; 4,752 participants | Low-PD-L1 focus; IPD reconstruction | Did not provide the present 14-family/31-report linkage and 21-model multiplicity family | Adds post-2021 trial families and later long-term, regional, correction, and biomarker reports; extends beyond a low-PD-L1 focus. |
| Zafar et al., 2024 (PMID 38205080) | 6 Dec 2022 | 13 phase III trials; 6,519 participants | Mixed esophageal cancer; overall efficacy and ongoing trials | Broader histology/strategy scope; not the present strict trial-family RHR framework | Adds later trial families and report-level linkage; restricts mixed-histology trials to explicit ESCC/SCC estimates. |
| Beshr et al., 2025 (PMID 40769303) | 28 Feb 2025 | 13 papers; 6,672 participants | First- and later-line clinical and PD-L1 subgroups | Publication count differs from independent trial-family count; present review adds formal RHR/multiplicity | Extends the search to 1 August 2026 and adds later long-term and biomarker reports, including scoring-specific PD-L1 analyses. |
| Gao et al., 2026 (PMID 41861173) | Not reported in PubMed abstract | 8 RCTs; 4,702 participants | First-line PD-1 inhibitor plus chemotherapy; beneficial subgroups | First-line only; present review separates lines and tests interactions formally | Extends beyond first-line therapy and links 31 reports to 14 randomized trial families; the report-level increment cannot be determined from the abstracted search information. |
| Present review | 1 August 2026 | 14 trial families; 31 reports; primary OS 12 families/N = 6,121 | Trial-family deduplication; line separation; scoring-specific PD-L1; within-trial RHR; Holm/Bonferroni | No reliable modifier after multiplicity correction; sparse-evidence labels and conditional ASTRUM sensitivities | Fourteen trial families and 31 linked reports; treatment-line- and scoring-specific RHRs; patient de-duplication across updates; 21-model family-wise multiplicity control. |

## **Table S4. Linkage of reports to randomized trial families**

## **Table S4A.** Fourteen independent randomized trial families

| **Trial / NCT** | **Line** | **Histology scope** | **Trial N / strict ESCC N** | **Reports** | **Strategies** | **Analytical role** |
| --- | --- | --- | --- | --- | --- | --- |
| CheckMate 648 NCT03143153 | first line | ESCC only | 970 / 970 | 3 | PD-1 inhibitor plus chemotherapy; Dual checkpoint inhibition without chemotherapy | Nivolumab plus chemotherapy was included in the primary first-line analysis; nivolumab plus ipilimumab was analyzed separately. The shared chemotherapy control was counted once per synthesis. |
| KEYNOTE-590 NCT03189719 | first line | mixed ESCC and adenocarcinoma | 749 / 548 | 3 | PD-1 inhibitor plus chemotherapy | Only estimates reported explicitly for ESCC were included. |
| ESCORT-1st NCT03691090 | first line | ESCC only | 596 / 596 | 2 | PD-1 inhibitor plus chemotherapy | Included in the first-line single-checkpoint analysis. |
| GEMSTONE-304 NCT04187352 | first line | ESCC only | 540 / 540 | 1 | PD-L1 inhibitor plus chemotherapy | Included in the first-line single-checkpoint analysis. |
| SKYSCRAPER-08 NCT04540211 | first line | ESCC only | 461 / 461 | 1 | Dual checkpoint inhibition plus chemotherapy | Analyzed separately as a dual-checkpoint strategy and excluded from the primary single-checkpoint model. |
| ASTRUM-007 NCT03958890 | first line | ESCC only; PD-L1 CPS ≥ 1 enriched | 551 / 551 | 2 | PD-1 inhibitor plus chemotherapy | Included only in CPS ≥ 1-enriched sensitivity analyses and excluded from primary all-comer models. |
| JUPITER-06 NCT03829969 | first line | ESCC only | 514 / 514 | 3 | PD-1 inhibitor plus chemotherapy | Included in the first-line single-checkpoint analysis. |
| ORIENT-15 NCT03748134 | first line | ESCC only | 659 / 659 | 1 | PD-1 inhibitor plus chemotherapy | Included in the first-line single-checkpoint analysis. |
| RATIONALE-306 NCT03783442 | first line | ESCC only | 649 / 649 | 2 | PD-1 inhibitor plus chemotherapy | Included in the first-line single-checkpoint analysis. |
| ATTRACTION-3 NCT02569242 | second or later line | ESCC only | 419 / 419 | 4 | PD-1 monotherapy versus investigator-choice taxane chemotherapy | Included in the later-line single-checkpoint analysis. |
| ESCORT NCT03099382 | second or later line | ESCC only | 457 / 457 | 1 | PD-1 monotherapy versus investigator-choice chemotherapy | Included in the later-line single-checkpoint analysis. |
| ORIENT-2 NCT03116152 | second or later line | ESCC only | 190 / 190 | 1 | PD-1 monotherapy versus chemotherapy | Included in the later-line single-checkpoint analysis. |
| KEYNOTE-181 NCT02564263 | second or later line | Mixed esophageal squamous cell carcinoma and adenocarcinoma in the global trial; only explicitly SCC/ESCC-restricted rows are eligible here | 628 / 401 | 3 | PD-1 monotherapy versus investigator-choice chemotherapy | Only estimates reported explicitly for SCC/ESCC were included. |
| RATIONALE-302 NCT03430843 | second or later line | ESCC only | 512 / 512 | 4 | PD-1 monotherapy versus investigator-choice chemotherapy | Included in the later-line single-checkpoint analysis. |

## **Table S4B.** Thirty-one linked reports

| **Trial family** | **PMID** | **Year** | **Report type** | **Data cutoff** | **Population scope** | **Selection/linkage role** |
| --- | --- | --- | --- | --- | --- | --- |
| CheckMate 648 | 35108470 | 2022 | Primary publication with supplementary appendix | Not reported | full ESCC ITT | Used for data extraction |
| CheckMate 648 | 38716626 | 2024 | long-term update | 2022-05-17 | full ESCC ITT | Used for data extraction |
| CheckMate 648 | 39927664 | 2025 | correction | Not reported | correction notice | Correction or erratum; no separate numerical estimate |
| KEYNOTE-590 | 34454674 | 2021 | primary publication | Not reported | mixed histology; selected ESCC efficacy cohorts reported | No numerical HR extracted; linked to the corresponding trial family |
| KEYNOTE-590 | 34801105 | 2021 | correction | Not reported | correction notice | Correction or erratum; no separate numerical estimate |
| KEYNOTE-590 | 41259897 | 2025 | 5-year update | Not reported | mixed histology with explicit ESCC and ESCC-by-CPS rows | Used for data extraction |
| ESCORT-1st | 34519801 | 2021 | Primary publication with supplementary appendix, protocol, and statistical analysis plan | 2020-10-30 | full ESCC ITT | Used for data extraction |
| ESCORT-1st | 38870932 | 2024 | Final overall-survival report with supplementary appendix, protocol, and statistical analysis plan | 2022-04-30 | full ESCC ITT | Used for data extraction |
| GEMSTONE-304 | 38302715 | 2024 | primary publication | 2022-10-07 | full ESCC ITT | Used for data extraction |
| SKYSCRAPER-08 | 41449142 | 2026 | primary/final OS publication | 2023-02-13 | full ESCC ITT | Not used in quantitative synthesis; linked to the corresponding trial family |
| ASTRUM-007 | 36732627 | 2023 | primary report | 2022-04-15 | randomized ITT ESCC population, all with PD-L1 CPS ≥ 1 | Used for data extraction |
| ASTRUM-007 | 38769930 | 2024 | post-hoc subgroup report with article-level meta-analysis | 2023-01-09 | ASTRUM-007 randomized ITT ESCC population, all with PD-L1 CPS ≥ 1; liver-metastasis post-hoc analysis | Used for data extraction |
| JUPITER-06 | 35245446 | 2022 | primary report | 2021-03-22 | randomized ITT ESCC population | Not used in quantitative synthesis; linked to the corresponding trial family |
| JUPITER-06 | 36473145 | 2023 | post-hoc PD-L1 report with article-level meta-analysis | 2021-03-22 | JUPITER-06 randomized ITT ESCC cohort with evaluable PD-L1 TPS | Used for data extraction |
| JUPITER-06 | 41571044 | 2026 | final OS and biomarker update | 2023-02-23 | randomized ITT ESCC population | Used for data extraction |
| ORIENT-15 | 35440464 | 2022 | primary report | 2021-04-09 | randomized ITT ESCC population | Used for data extraction |
| RATIONALE-306 | 37080222 | 2023 | primary report | 2022-02-28 | randomized all-comer ITT ESCC population | Used for data extraction |
| RATIONALE-306 | 40075024 | 2025 | retrospective PD-L1 TAP ≥ 1% subgroup analysis | 2022-02-28 primary analysis; 2023-11-24 final analysis | baseline PD-L1 TAP ≥ 1% subgroup only; not the all-comer RATIONALE-306 ITT population | Used for data extraction |
| ATTRACTION-3 | 31582355 | 2019 | primary randomized trial report; corrected online version | 2018-11-12 | Global ITT population, n=419; all ESCC | Used for data extraction |
| ATTRACTION-3 | 31674317 | 2019 | published correction / erratum | Not reported | Correction to A3_PRIMARY_2019; no new patient analysis | Correction or erratum; no separate numerical estimate |
| ATTRACTION-3 | 33170461 | 2021 | regional subgroup report | 2018-11-12 | Japanese randomized subset n=274 (nivolumab 136; chemotherapy 138), wholly nested within global ATTRACTION-3 n=419 | Nested regional subgroup used only in sensitivity or descriptive analyses |
| ATTRACTION-3 | 35294546 | 2022 | long-term follow-up and exploratory response-survival analysis | 2020-05-25 | Same global ITT cohort, n=419; all ESCC | Used for data extraction |
| ESCORT | 32416073 | 2020 | primary randomized trial report | 2019-05-06 | Chinese randomized ESCC cohort; OS full-analysis set n=448 | Used for data extraction |
| ORIENT-2 | 35165274 | 2022 | primary randomized trial report with exploratory biomarker analyses | 2019-08-02 | Chinese randomized ITT population, n=190; all ESCC | Used for data extraction |
| KEYNOTE-181 | 33026938 | 2020 | primary randomized trial report with embedded data supplement | 2018-10-15 (updated analysis adds two events); 2019-02-13 additional follow-up | Global mixed-histology ITT n=628; Figure 3B explicitly restricts the target rows to squamous cell carcinoma n=401 | Used for data extraction |
| KEYNOTE-181 | 34973513 | 2022 | post hoc Asian ESCC subgroup plus China-extension pooled analysis | 2018-10-15 for global KEYNOTE-181; 2019-02-13 for China extension | Pooled Asian ESCC n=340: 221 non-Chinese Asian patients from the global trial plus a China cohort n=119 (10 global-trial Chinese + 109 China-extension ESCC) | Used for data extraction |
| KEYNOTE-181 | 34591237 | 2022 | Japanese regional subgroup report | 2019-02-13 for Figure 2 | Japanese randomized subset n=152, wholly nested within global KEYNOTE-181; only two explicit SCC/ESCC Figure 2 rows were extracted | nested regional strict-ESCC sensitivity/descriptive records; not an independent RCT |
| RATIONALE-302 | 35442766 | 2022 | Primary randomized trial report with data supplement, protocol, and statistical analysis plan | 2020-12-01 | Global ITT population n=512; all ESCC | Used for data extraction |
| RATIONALE-302 | 38113437 | 2023 | published erratum | Not reported | Correction to R302_PRIMARY_2022; no new patient analysis | Correction or erratum; no separate numerical estimate |
| RATIONALE-302 | 38118368 | 2024 | Europe/North America regional subgroup report with supplementary appendix | 2020-12-01 | Europe/North America randomized subset n=108 (55 vs 53); fully nested in the global trial | Not used in quantitative synthesis; linked to the corresponding trial family |
| RATIONALE-302 | 38240916 | 2024 | Japanese regional subgroup report | 2020-12-01 | Japanese randomized subset n=50 (25 versus 25), wholly nested within global RATIONALE-302 n=512 | Nested regional subgroup used only in sensitivity or descriptive analyses |

## **Table S5.** Detailed trial-family characteristics, PD-L1 definitions, and analytical roles

## **Table S5A.** Trial design and randomized interventions

| **Trial / registration** | **Phase / line** | **Region** | **Histology** | **Randomized population / arms** | **Experimental regimen** | **Control regimen** |
| --- | --- | --- | --- | --- | --- | --- |
| CheckMate 648 NCT03143153 | Phase III First line | Global; 182 sites in 26 countries | ESCC only | 970 Nivolumab plus chemotherapy n=321; nivolumab plus ipilimumab n=325; chemotherapy n=324 | Nivolumab plus fluorouracil/cisplatin; separate arm nivolumab plus ipilimumab | Fluorouracil/cisplatin chemotherapy |
| KEYNOTE-590 NCT03189719 | Phase III First line | Global; 168 centers in 26 countries | Mixed esophageal/GEJ histology; ESCC-specific estimates only are eligible | Overall n=749 (pembrolizumab arm n=373; control n=376); ESCC n=548 (274/274) Pembrolizumab plus chemotherapy n=373; placebo plus chemotherapy n=376; within ESCC, 274/274 | Pembrolizumab plus cisplatin/5-fluorouracil | Placebo plus cisplatin/5-fluorouracil |
| ESCORT-1st NCT03691090 | Phase III First line | China; 60 hospitals | ESCC only | 596 Camrelizumab plus chemotherapy n=298; placebo plus chemotherapy n=298 | Camrelizumab plus paclitaxel/cisplatin | Placebo plus paclitaxel/cisplatin |
| GEMSTONE-304 NCT04187352 | Phase III First line | China | ESCC only | 540 Sugemalimab plus chemotherapy n=358; placebo plus chemotherapy n=182 | Sugemalimab plus cisplatin/5-fluorouracil | Placebo plus cisplatin/5-fluorouracil |
| SKYSCRAPER-08 NCT04540211 | Phase III First line | Asia: mainland China, South Korea, Thailand, Taiwan, and Hong Kong | ESCC only | 461 Tiragolumab plus atezolizumab plus chemotherapy n=229; placebo plus chemotherapy n=232 | Tiragolumab plus atezolizumab plus paclitaxel/cisplatin | Placebo plus paclitaxel/cisplatin |
| ASTRUM-007 NCT03958890 | Phase III First line | China | ESCC only; enrollment restricted to PD-L1 CPS ≥ 1 | 551 Serplulimab plus chemotherapy n=368; placebo plus chemotherapy n=183 | Serplulimab plus cisplatin/5-fluorouracil | Placebo plus cisplatin/5-fluorouracil |
| JUPITER-06 NCT03829969 | Phase III First line | China | ESCC only | 514 Toripalimab plus chemotherapy n=257; placebo plus chemotherapy n=257 | Toripalimab plus paclitaxel/cisplatin | Placebo plus paclitaxel/cisplatin |
| ORIENT-15 NCT03748134 | Phase III First line | Multiregional: 66 sites in China and 13 sites outside China; predominantly Chinese enrollment | ESCC only | 659 Sintilimab plus chemotherapy n=327; placebo plus chemotherapy n=332 | Sintilimab plus investigator-chosen chemotherapy | Placebo plus investigator-chosen chemotherapy |
| RATIONALE-306 NCT03783442 | Phase III First line | Global; 162 centers across Asia, Europe, Oceania, and North America | ESCC only; all-comer population | 649 Tislelizumab plus chemotherapy n=326; placebo plus chemotherapy n=323 | Tislelizumab plus investigator-chosen chemotherapy doublet | Placebo plus investigator-chosen chemotherapy doublet |
| ATTRACTION-3 NCT02569242 | Phase III Second or later line | Denmark, Germany, Italy, Japan, South Korea, Taiwan, United Kingdom, and United States | ESCC only | 419 Nivolumab n=210; investigator-choice chemotherapy n=209 | Nivolumab 240 mg every 2 weeks | Investigator-choice paclitaxel or docetaxel |
| ESCORT NCT03099382 | Phase III Second or later line | China; 43 hospitals | ESCC only | Randomized n=457 (229/228); primary OS full-analysis set n=448 (228/220) Randomized: camrelizumab n=229, chemotherapy n=228; OS full-analysis set: camrelizumab n=228, chemotherapy n=220 | Camrelizumab 200 mg every 2 weeks | Investigator-choice docetaxel or irinotecan |
| ORIENT-2 NCT03116152 | Phase II Second or later line | China | ESCC only | 190 Sintilimab n=95; chemotherapy n=95 | Sintilimab 200 mg every 3 weeks | Paclitaxel or irinotecan |
| KEYNOTE-181 NCT02564263 | Phase III Second or later line | Global; 154 sites in 32 countries; later Asian report includes a China extension | Mixed squamous cell carcinoma and adenocarcinoma; only explicit SCC/ESCC rows are eligible | Overall n=628 (314/314). At the 2018-10-15 target cutoff, SCC n=401 (pembrolizumab 198; chemotherapy 203); at 2019-02-13, classification update yielded SCC n=403 (199/204) Overall: pembrolizumab n=314, chemotherapy n=314; target SCC Figure 3B: 198/203 at 2018-10-15 | Pembrolizumab 200 mg every 3 weeks | Investigator-choice paclitaxel, docetaxel, or irinotecan |
| RATIONALE-302 NCT03430843 | Phase III Second or later line | Global; 11 countries/regions across Asia, Europe, and North America | ESCC only | 512 Tislelizumab n=256; chemotherapy n=256 | Tislelizumab 200 mg every 3 weeks | Investigator-choice paclitaxel, docetaxel, or irinotecan |

## **Table S5B. PD-L1 definitions and linked reports by trial family**

| **Trial / registration** | **PD-L1 assay and scoring framework** | **Primary report / data cutoff** | **All linked PMIDs** | **Selected analysis source(s)** | **Analytical role and deduplication** |
| --- | --- | --- | --- | --- | --- |
| CheckMate 648 NCT03143153 | Dako/Agilent PD-L1 IHC 28-8 pharmDx; clone 28-8; Tumor-cell PD-L1 percentage in the primary endpoint framework; CPS also reported in exploratory analyses | PMID 35108470 (2022) Cutoff: Not explicitly stated in the primary report; minimum follow-up 13 months | 35108470, 38716626, 39927664 | PMID 35108470 primary report; PMID 38716626 29-month update, cutoff 2022-05-17 | The nivolumab-plus-chemotherapy contrast was used in the primary single-checkpoint analysis; nivolumab plus ipilimumab was analyzed separately. The shared chemotherapy control was not entered twice in the same pairwise model. |
| KEYNOTE-590 NCT03189719 | PD-L1 IHC 22C3 assay; clone 22C3; Combined positive score (CPS) | PMID 34454674 (2021) Cutoff: 2020-07-02 | 34454674, 34801105, 41259897 | PMID 41259897 5-year update, database cutoff 2023-07-10 | Only estimates explicitly reported for ESCC were eligible; subgroup estimates for the mixed-histology trial population were excluded. |
| ESCORT-1st NCT03691090 | SAB028 PD-L1 IHC using clone 6E8; Tumor proportion score (TPS) | PMID 34519801 (2021) Cutoff: 2020-10-30 | 34519801, 38870932 | Clinical subgroups from PMID 34519801, cutoff 2020-10-30; final PD-L1 report PMID 38870932, cutoff 2022-04-30 | Eligible first-line single-checkpoint trial. Primary and final reports represent the same cohort; one report was selected for each factor and cutoff. |
| GEMSTONE-304 NCT04187352 | VENTANA SP263 validated assay on BenchMark ULTRA; clone SP263; Study-specific percentage incorporating PD-L1-positive tumor cells and mononuclear inflammatory cells relative to viable tumor; the report does not label it standard CPS, TPS, or TAP | PMID 38302715 (2024) Cutoff: 2022-10-07 | 38302715 | PMID 38302715, cutoff 2022-10-07 | Eligible first-line single-checkpoint trial; the anti-PD-L1 classification was retained. One report represented this randomized cohort. |
| SKYSCRAPER-08 NCT04540211 | VENTANA PD-L1 SP263 assay; clone SP263; Tumor area positivity (TAP) | PMID 41449142 (2026) Cutoff: 2023-02-13 | 41449142 | PMID 41449142, cutoff 2023-02-13 | Analyzed separately as a dual-checkpoint-plus-chemotherapy strategy and excluded from the primary single-checkpoint pool. |
| ASTRUM-007 NCT03958890 | PD-L1 IHC 22C3 pharmDx; clone 22C3; Combined positive score (CPS); trial eligibility required CPS ≥ 1 | PMID 36732627 (2023) Cutoff: 2022-04-15 | 36732627, 38769930 | Primary clinical/PD-L1 report PMID 36732627, cutoff 2022-04-15; liver-metastasis post hoc report PMID 38769930, cutoff 2023-01-09 | Because eligibility required CPS ≥ 1, the trial was excluded from primary all-comer OS and clinical-interaction analyses and included only in enriched or conditional sensitivity analyses. Six clinical RHRs were not included in the 21-comparison multiplicity adjustment; smoking status was not estimable without ASTRUM-007. The liver-metastasis report represented the same randomized cohort, and only its trial-specific estimate was used. |
| JUPITER-06 NCT03829969 | PD-L1 IHC with clone JS311 on VENTANA BenchMark ULTRA; CPS and TPS reported in different analyses | PMID 35245446 (2022) Cutoff: 2021-03-22 | 35245446, 36473145, 41571044 | Clinical/CPS update PMID 41571044, cutoff 2023-02-23; TPS post hoc report PMID 36473145 used the 2021-03-22 cutoff | Eligible first-line single-checkpoint trial. Primary, PD-L1, and final reports represent the same cohort; one compatible estimate was selected for each factor and scoring system. |
| ORIENT-15 NCT03748134 | PD-L1 IHC 22C3 pharmDx; clone 22C3; CPS and TPS | PMID 35440464 (2022) Cutoff: 2021-04-09 | 35440464 | PMID 35440464, cutoff 2021-04-09 | Eligible first-line single-checkpoint trial. The outside-China subgroup HR was not estimable and was not imputed. |
| RATIONALE-306 NCT03783442 | VENTANA PD-L1 SP263 assay/CDx research-use version; clone SP263; Tumor area positivity (TAP) | PMID 37080222 (2023) Cutoff: 2022-02-28 | 37080222, 40075024 | All-comer clinical/PD-L1 estimates from PMID 37080222, cutoff 2022-02-28; TAP ≥ 1 post-hoc report PMID 40075024 also reports final cutoff 2023-11-24 | Eligible first-line single-checkpoint trial. TAP ≥ 1 conditional analyses were analyzed separately and did not replace all-comer ITT clinical estimates. |
| ATTRACTION-3 NCT02569242 | Central Dako/Agilent PD-L1 IHC 28-8 pharmDx; clone 28-8; Tumor-cell PD-L1 expression / tumor proportion percentage; thresholds 1%, 5%, and 10% | PMID 31582355 (2019) Cutoff: 2018-11-12 | 31582355, 31674317, 33170461, 35294546 | Primary clinical report PMID 31582355, cutoff 2018-11-12; 3-year update PMID 35294546, cutoff 2020-05-25 | Eligible later-line trial. Regional results were used only for sensitivity or descriptive analyses because they were nested in the global cohort. |
| ESCORT NCT03099382 | Human PD-L1 IHC kit from Shuwen Biotech; clone 6E8; Tumor-cell PD-L1 expression / tumor proportion score | PMID 32416073 (2020) Cutoff: 2019-05-06 | 32416073 | PMID 32416073, cutoff 2019-05-06 | Eligible later-line trial. The randomized population (n = 457) was distinguished from the treated/full-analysis OS population (n = 448). |
| ORIENT-2 NCT03116152 | PD-L1 IHC 22C3 pharmDx (Dako/Agilent); clone 22C3; CPS and TPS | PMID 35165274 (2022) Cutoff: 2019-08-02 | 35165274 | PMID 35165274, cutoff 2019-08-02 | Eligible later-line trial. Biomarker analyses confined to the sintilimab arm were not used as between-arm treatment-effect subgroups. |
| KEYNOTE-181 NCT02564263 | PD-L1 IHC 22C3 pharmDx (Agilent); clone 22C3; Combined positive score (CPS) | PMID 33026938 (2020) Cutoff: 2018-10-15 for the target SCC subgroup forest; additional follow-up 2019-02-13 | 33026938, 34973513, 34591237 | Global SCC rows from PMID 33026938; Asian pooled report PMID 34973513 combines global and China-extension data and must replace, not duplicate, the global family contribution | Only estimates explicitly restricted to SCC/ESCC were eligible. The pooled Asian report included participants from the global trial and the China-extension cohort and therefore replaced, rather than supplemented, the global trial-family contribution. The Japanese report was used only for strict-ESCC sensitivity analyses. |
| RATIONALE-302 NCT03430843 | VENTANA PD-L1 SP263 assay; clone SP263; Parent trial: tumor area positivity (TAP). Japanese report uses the source term visually estimated combined positive score (vCPS), which must not be silently converted to standard 22C3 CPS | PMID 35442766 (2022) Cutoff: 2020-12-01 | 35442766, 38113437, 38118368, 38240916 | Global primary report PMID 35442766, cutoff 2020-12-01; Europe/North America and Japanese regional reports use the same parent-trial cutoff | Eligible later-line trial. The vCPS definition in the Japanese report was retained as reported and was not treated as standard 22C3 CPS. Regional reports were used only for sensitivity or descriptive analyses because they were nested in the global cohort. |

## Table S6. Extracted PD-L1 subgroup estimates and source locations

| **Trial** | **Line** | **PMID** | **Scoring system** | **Cutoff** | **Category** | **N** | **HR (95% CI)** | **Analysis role** | **Trial-family/overlap note** | **Exact source location** |
| --- | --- | --- | --- | --- | --- | --- | --- | --- | --- | --- |
| CheckMate 648 | first line | 35108470 | tumor-cell PD-L1 percentage | 1% | ≥1% | 314 | 0.550 (0.420–0.720) | Included in a scoring-system- and cutoff-specific interaction analysis; one compatible estimate per trial family | Multiple PD-L1 cutoffs from the same trial are correlated; each model used one prespecified cutoff or contrast per trial family. | PMID 35108470, Supplementary Figure S3B |
| CheckMate 648 | first line | 35108470 | tumor-cell PD-L1 percentage | 1% | <1% | 329 | 0.980 (0.760–1.280) | Included in a scoring-system- and cutoff-specific interaction analysis; one compatible estimate per trial family | Multiple PD-L1 cutoffs from the same trial are correlated; each model used one prespecified cutoff or contrast per trial family. | PMID 35108470, Supplementary Figure S3B |
| CheckMate 648 | first line | 35108470 | tumor-cell PD-L1 percentage | 5% | ≥5% | 235 | 0.610 (0.450–0.830) | Secondary cutoff or category reported descriptively; not included in multiplicity-adjusted analyses | Multiple PD-L1 cutoffs from the same trial are correlated; each model used one prespecified cutoff or contrast per trial family. | PMID 35108470, Supplementary Figure S3B |
| CheckMate 648 | first line | 35108470 | tumor-cell PD-L1 percentage | 5% | <5% | 408 | 0.820 (0.650–1.040) | Secondary cutoff or category reported descriptively; not included in multiplicity-adjusted analyses | Multiple PD-L1 cutoffs from the same trial are correlated; each model used one prespecified cutoff or contrast per trial family. | PMID 35108470, Supplementary Figure S3B |
| CheckMate 648 | first line | 35108470 | tumor-cell PD-L1 percentage | 10% | ≥10% | 199 | 0.620 (0.440–0.870) | Secondary cutoff or category reported descriptively; not included in multiplicity-adjusted analyses | Multiple PD-L1 cutoffs from the same trial are correlated; each model used one prespecified cutoff or contrast per trial family. | PMID 35108470, Supplementary Figure S3B |
| CheckMate 648 | first line | 35108470 | tumor-cell PD-L1 percentage | 10% | <10% | 444 | 0.790 (0.630–0.990) | Secondary cutoff or category reported descriptively; not included in multiplicity-adjusted analyses | Multiple PD-L1 cutoffs from the same trial are correlated; each model used one prespecified cutoff or contrast per trial family. | PMID 35108470, Supplementary Figure S3B |
| CheckMate 648 | first line | 35108470 | CPS | 1 | ≥1 | 558 | 0.690 (0.560–0.840) | Included in a scoring-system- and cutoff-specific interaction analysis; one compatible estimate per trial family | Multiple PD-L1 cutoffs from the same trial are correlated; each model used one prespecified cutoff or contrast per trial family. | PMID 35108470, Supplementary Figure S3B |
| CheckMate 648 | first line | 35108470 | CPS | 1 | <1 | 51 | 0.980 (0.500–1.950) | Included in a scoring-system- and cutoff-specific interaction analysis; one compatible estimate per trial family | Multiple PD-L1 cutoffs from the same trial are correlated; each model used one prespecified cutoff or contrast per trial family. | PMID 35108470, Supplementary Figure S3B |
| CheckMate 648 | first line | 35108470 | CPS | 5 | ≥5 | 421 | 0.690 (0.550–0.870) | Secondary cutoff or category reported descriptively; not included in multiplicity-adjusted analyses | Multiple PD-L1 cutoffs from the same trial are correlated; each model used one prespecified cutoff or contrast per trial family. | PMID 35108470, Supplementary Figure S3B |
| CheckMate 648 | first line | 35108470 | CPS | 5 | <5 | 188 | 0.740 (0.520–1.040) | Secondary cutoff or category reported descriptively; not included in multiplicity-adjusted analyses | Multiple PD-L1 cutoffs from the same trial are correlated; each model used one prespecified cutoff or contrast per trial family. | PMID 35108470, Supplementary Figure S3B |
| CheckMate 648 | first line | 35108470 | CPS | 10 | ≥10 | 280 | 0.630 (0.470–0.840) | Included in a scoring-system- and cutoff-specific interaction analysis; one compatible estimate per trial family | Multiple PD-L1 cutoffs from the same trial are correlated; each model used one prespecified cutoff or contrast per trial family. | PMID 35108470, Supplementary Figure S3B |
| CheckMate 648 | first line | 35108470 | CPS | 10 | <10 | 329 | 0.780 (0.600–1.010) | Included in a scoring-system- and cutoff-specific interaction analysis; one compatible estimate per trial family | Multiple PD-L1 cutoffs from the same trial are correlated; each model used one prespecified cutoff or contrast per trial family. | PMID 35108470, Supplementary Figure S3B |
| KEYNOTE-590 | first line | 41259897 | CPS | 1 | ≥1 | 478 | 0.690 (0.570–0.840) | Included in a scoring-system- and cutoff-specific interaction analysis; one compatible estimate per trial family | The long-term update represented the same trial family; compatible CPS estimates replaced those from the earlier report. | PMID 41259897, Figure 2D and Table 2 |
| KEYNOTE-590 | first line | 41259897 | CPS | 10 | ≥10 | 286 | 0.600 (0.460–0.760) | Included in a scoring-system- and cutoff-specific interaction analysis; one compatible estimate per trial family | The long-term update represented the same trial family; compatible CPS estimates replaced those from the earlier report. | PMID 41259897, Figure 2D and Table 2 |
| KEYNOTE-590 | first line | 41259897 | CPS | 1 | <1 | 55 | 1.050 (0.590–1.850) | Included in a scoring-system- and cutoff-specific interaction analysis; one compatible estimate per trial family | The long-term update represented the same trial family; compatible CPS estimates replaced those from the earlier report. | PMID 41259897, Figure 2D and Table 2 |
| KEYNOTE-590 | first line | 41259897 | CPS | 10 | <10 | 247 | 0.900 (0.690–1.180) | Included in a scoring-system- and cutoff-specific interaction analysis; one compatible estimate per trial family | The long-term update represented the same trial family; compatible CPS estimates replaced those from the earlier report. | PMID 41259897, Figure 2D and Table 2 |
| ESCORT-1st | first line | 34519801 | TPS | 1% | <1% | 256 | 0.790 (0.570–1.110) | Included in a scoring-system- and cutoff-specific interaction analysis; one compatible estimate per trial family | The later data-cutoff report represented the same trial family and was not counted as an independent RCT. | PMID 34519801, eFigure 4 |
| ESCORT-1st | first line | 34519801 | TPS | 1% | ≥1% | 329 | 0.590 (0.430–0.800) | Included in a scoring-system- and cutoff-specific interaction analysis; one compatible estimate per trial family | The later data-cutoff report represented the same trial family and was not counted as an independent RCT. | PMID 34519801, eFigure 4 |
| ESCORT-1st | first line | 34519801 | TPS | 5% | <5% | 300 | 0.770 (0.560–1.040) | Secondary cutoff or category reported descriptively; not included in multiplicity-adjusted analyses | The later data-cutoff report represented the same trial family and was not counted as an independent RCT. | PMID 34519801, eFigure 4 |
| ESCORT-1st | first line | 34519801 | TPS | 5% | ≥5% | 285 | 0.600 (0.430–0.840) | Secondary cutoff or category reported descriptively; not included in multiplicity-adjusted analyses | The later data-cutoff report represented the same trial family and was not counted as an independent RCT. | PMID 34519801, eFigure 4 |
| ESCORT-1st | first line | 34519801 | TPS | 10% | <10% | 383 | 0.780 (0.590–1.020) | Secondary cutoff or category reported descriptively; not included in multiplicity-adjusted analyses | The later data-cutoff report represented the same trial family and was not counted as an independent RCT. | PMID 34519801, eFigure 4 |
| ESCORT-1st | first line | 34519801 | TPS | 10% | ≥10% | 202 | 0.520 (0.350–0.790) | Secondary cutoff or category reported descriptively; not included in multiplicity-adjusted analyses | The later data-cutoff report represented the same trial family and was not counted as an independent RCT. | PMID 34519801, eFigure 4 |
| ESCORT-1st | first line | 38870932 | TPS | <1% | TPS <1% | 256 | 0.770 (0.590–1.020) | Secondary cutoff or category reported descriptively; not included in multiplicity-adjusted analyses | The later data cutoff represented the same randomized cohort and replaced overlapping estimates from the primary report. | PMID 38870932, Table 3 |
| ESCORT-1st | first line | 38870932 | TPS | ≥1–<10% | TPS ≥1% to <10% | 127 | 0.670 (0.450–1.000) | Secondary cutoff or category reported descriptively; not included in multiplicity-adjusted analyses | The later data cutoff represented the same randomized cohort and replaced overlapping estimates from the primary report. | PMID 38870932, Table 3 |
| ESCORT-1st | first line | 38870932 | TPS | ≥10–<50% | TPS ≥10% to <50% | 153 | 0.570 (0.400–0.830) | Secondary cutoff or category reported descriptively; not included in multiplicity-adjusted analyses | The later data cutoff represented the same randomized cohort and replaced overlapping estimates from the primary report. | PMID 38870932, Table 3 |
| ESCORT-1st | first line | 38870932 | TPS | ≥50% | TPS ≥50% | 49 | 0.750 (0.350–1.630) | Secondary cutoff or category reported descriptively; not included in multiplicity-adjusted analyses | The later data cutoff represented the same randomized cohort and replaced overlapping estimates from the primary report. | PMID 38870932, Table 3 |
| GEMSTONE-304 | first line | 38302715 | study-specific percentage of PD-L1-positive tumor cells and mononuclear inflammatory cells relative to viable tumor cells; not explicitly named CPS/TPS/TAP | <1% | <1% | 62 | 0.630 (0.320–1.240) | Secondary cutoff or category reported descriptively; not included in multiplicity-adjusted analyses | Multiple PD-L1 cutoffs from the same trial are correlated; each model used one prespecified cutoff or contrast per trial family. | PMID 38302715, Figure 3a |
| GEMSTONE-304 | first line | 38302715 | study-specific percentage of PD-L1-positive tumor cells and mononuclear inflammatory cells relative to viable tumor cells; not explicitly named CPS/TPS/TAP | 1–<10% | ≥1% and <10% | 246 | 0.920 (0.640–1.320) | Secondary cutoff or category reported descriptively; not included in multiplicity-adjusted analyses | Multiple PD-L1 cutoffs from the same trial are correlated; each model used one prespecified cutoff or contrast per trial family. | PMID 38302715, Figure 3a |
| GEMSTONE-304 | first line | 38302715 | study-specific percentage of PD-L1-positive tumor cells and mononuclear inflammatory cells relative to viable tumor cells; not explicitly named CPS/TPS/TAP | ≥10% | ≥10% | 232 | 0.570 (0.390–0.830) | Secondary cutoff or category reported descriptively; not included in multiplicity-adjusted analyses | Multiple PD-L1 cutoffs from the same trial are correlated; each model used one prespecified cutoff or contrast per trial family. | PMID 38302715, Figure 3a |
| ASTRUM-007 | first line | 36732627 | CPS | 1 <= CPS < 10 | 1 <= CPS < 10 | 310 | 0.740 (0.540–1.030) | CPS ≥ 1-enriched secondary analysis; not included in all-comer or multiplicity-adjusted analyses | Enrollment was restricted to CPS ≥ 1, so no CPS < 1 comparator was available; the estimate was treated as enriched evidence from the same trial family. | PMID 36732627, Figure 4b |
| ASTRUM-007 | first line | 36732627 | CPS | 10 | CPS > =10 | 241 | 0.590 (0.400–0.880) | CPS ≥ 1-enriched secondary analysis; not included in all-comer or multiplicity-adjusted analyses | Enrollment was restricted to CPS ≥ 1, so no CPS < 1 comparator was available; the estimate was treated as enriched evidence from the same trial family. | PMID 36732627, Figure 4b |
| JUPITER-06 | first line | 36473145 | TPS | 1% | TPS >=1% | 295 | 0.610 (0.420–0.900) | Included in a scoring-system- and cutoff-specific interaction analysis; one compatible estimate per trial family | This post hoc PD-L1 report used the same randomized cohort; only the compatible TC/TPS cutoff was used in the corresponding model. | PMID 36473145, Figure 1C-E |
| JUPITER-06 | first line | 36473145 | TPS | 1% | TPS <1% | 193 | 0.630 (0.370–1.080) | Included in a scoring-system- and cutoff-specific interaction analysis; one compatible estimate per trial family | This post hoc PD-L1 report used the same randomized cohort; only the compatible TC/TPS cutoff was used in the corresponding model. | PMID 36473145, Figure 1C-E |
| JUPITER-06 | first line | 36473145 | TPS | 5% | TPS <5% | 318 | 0.660 (0.450–0.970) | Secondary cutoff or category reported descriptively; not included in multiplicity-adjusted analyses | This post hoc PD-L1 report used the same randomized cohort; only the compatible TC/TPS cutoff was used in the corresponding model. | PMID 36473145, Figure 1C-E |
| JUPITER-06 | first line | 36473145 | TPS | 5% | TPS >=5% | 170 | 0.570 (0.330–0.970) | Secondary cutoff or category reported descriptively; not included in multiplicity-adjusted analyses | This post hoc PD-L1 report used the same randomized cohort; only the compatible TC/TPS cutoff was used in the corresponding model. | PMID 36473145, Figure 1C-E |
| JUPITER-06 | first line | 36473145 | TPS | 10% | TPS <10% | 371 | 0.660 (0.460–0.930) | Secondary cutoff or category reported descriptively; not included in multiplicity-adjusted analyses | This post hoc PD-L1 report used the same randomized cohort; only the compatible TC/TPS cutoff was used in the corresponding model. | PMID 36473145, Figure 1C-E |
| JUPITER-06 | first line | 36473145 | TPS | 10% | TPS >=10% | 117 | 0.510 (0.260–1.020) | Secondary cutoff or category reported descriptively; not included in multiplicity-adjusted analyses | This post hoc PD-L1 report used the same randomized cohort; only the compatible TC/TPS cutoff was used in the corresponding model. | PMID 36473145, Figure 1C-E |
| JUPITER-06 | first line | 41571044 | CPS | 1 | CPS > =1 | 401 | 0.730 (0.580–0.920) | Included in a scoring-system- and cutoff-specific interaction analysis; one compatible estimate per trial family | The final biomarker update represented the same trial family and replaced earlier matching CPS estimates. | PMID 41571044, Figure 2B |
| JUPITER-06 | first line | 41571044 | CPS | 1 | CPS < 1 | 87 | 0.900 (0.550–1.480) | Included in a scoring-system- and cutoff-specific interaction analysis; one compatible estimate per trial family | The final biomarker update represented the same trial family and replaced earlier matching CPS estimates. | PMID 41571044, Figure 2B |
| JUPITER-06 | first line | 41571044 | CPS | 10 | CPS > =10 | 212 | 0.790 (0.570–1.100) | Included in a scoring-system- and cutoff-specific interaction analysis; one compatible estimate per trial family | The final biomarker update represented the same trial family and replaced earlier matching CPS estimates. | PMID 41571044, Figure 2B |
| JUPITER-06 | first line | 41571044 | CPS | 10 | CPS < 10 | 276 | 0.740 (0.560–0.980) | Included in a scoring-system- and cutoff-specific interaction analysis; one compatible estimate per trial family | The final biomarker update represented the same trial family and replaced earlier matching CPS estimates. | PMID 41571044, Figure 2B |
| ORIENT-15 | first line | 35440464 | CPS | 10 | CPS < 10 | 278 | 0.620 (0.450–0.850) | Included in a scoring-system- and cutoff-specific interaction analysis; one compatible estimate per trial family | Multiple PD-L1 cutoffs from the same trial are correlated; each model used one prespecified cutoff or contrast per trial family. | PMID 35440464, Figure 3 |
| ORIENT-15 | first line | 35440464 | CPS | 10 | CPS > =10 | 381 | 0.640 (0.480–0.850) | Included in a scoring-system- and cutoff-specific interaction analysis; one compatible estimate per trial family | Multiple PD-L1 cutoffs from the same trial are correlated; each model used one prespecified cutoff or contrast per trial family. | PMID 35440464, Figure 3 |
| ORIENT-15 | first line | 35440464 | CPS | 5 | CPS < 5 | 168 | 0.560 (0.370–0.860) | Secondary cutoff or category reported descriptively; not included in multiplicity-adjusted analyses | Multiple PD-L1 cutoffs from the same trial are correlated; each model used one prespecified cutoff or contrast per trial family. | PMID 35440464, Figure 3 |
| ORIENT-15 | first line | 35440464 | CPS | 5 | CPS > =5 | 491 | 0.650 (0.510–0.830) | Secondary cutoff or category reported descriptively; not included in multiplicity-adjusted analyses | Multiple PD-L1 cutoffs from the same trial are correlated; each model used one prespecified cutoff or contrast per trial family. | PMID 35440464, Figure 3 |
| ORIENT-15 | first line | 35440464 | CPS | 1 | CPS < 1 | 55 | 1.320 (0.630–2.770) | Included in a scoring-system- and cutoff-specific interaction analysis; one compatible estimate per trial family | Multiple PD-L1 cutoffs from the same trial are correlated; each model used one prespecified cutoff or contrast per trial family. | PMID 35440464, Figure 3 |
| ORIENT-15 | first line | 35440464 | CPS | 1 | CPS > =1 | 604 | 0.590 (0.470–0.740) | Included in a scoring-system- and cutoff-specific interaction analysis; one compatible estimate per trial family | Multiple PD-L1 cutoffs from the same trial are correlated; each model used one prespecified cutoff or contrast per trial family. | PMID 35440464, Figure 3 |
| ORIENT-15 | first line | 35440464 | TPS | 10% | TPS <10% | 421 | 0.670 (0.520–0.880) | Secondary cutoff or category reported descriptively; not included in multiplicity-adjusted analyses | Multiple PD-L1 cutoffs from the same trial are correlated; each model used one prespecified cutoff or contrast per trial family. | PMID 35440464, Figure 3 |
| ORIENT-15 | first line | 35440464 | TPS | 10% | TPS >=10% | 238 | 0.550 (0.380–0.780) | Secondary cutoff or category reported descriptively; not included in multiplicity-adjusted analyses | Multiple PD-L1 cutoffs from the same trial are correlated; each model used one prespecified cutoff or contrast per trial family. | PMID 35440464, Figure 3 |
| ORIENT-15 | first line | 35440464 | TPS | 5% | TPS <5% | 371 | 0.610 (0.460–0.820) | Secondary cutoff or category reported descriptively; not included in multiplicity-adjusted analyses | Multiple PD-L1 cutoffs from the same trial are correlated; each model used one prespecified cutoff or contrast per trial family. | PMID 35440464, Figure 3 |
| ORIENT-15 | first line | 35440464 | TPS | 5% | TPS >=5% | 288 | 0.670 (0.490–0.920) | Secondary cutoff or category reported descriptively; not included in multiplicity-adjusted analyses | Multiple PD-L1 cutoffs from the same trial are correlated; each model used one prespecified cutoff or contrast per trial family. | PMID 35440464, Figure 3 |
| ORIENT-15 | first line | 35440464 | TPS | 1% | TPS <1% | 297 | 0.550 (0.400–0.750) | Included in a scoring-system- and cutoff-specific interaction analysis; one compatible estimate per trial family | Multiple PD-L1 cutoffs from the same trial are correlated; each model used one prespecified cutoff or contrast per trial family. | PMID 35440464, Figure 3 |
| ORIENT-15 | first line | 35440464 | TPS | 1% | TPS >=1% | 362 | 0.710 (0.530–0.950) | Included in a scoring-system- and cutoff-specific interaction analysis; one compatible estimate per trial family | Multiple PD-L1 cutoffs from the same trial are correlated; each model used one prespecified cutoff or contrast per trial family. | PMID 35440464, Figure 3 |
| RATIONALE-306 | first line | 37080222 | TAP | 10% | TAP > =10% | 223 | 0.620 (0.440–0.870) | TAP subgroup reported descriptively; not included in multiplicity-adjusted analyses | TAP thresholds were derived from the same randomized cohort. TAP was analyzed separately from CPS and TC/TPS, and the single-trial contrast was reported descriptively. | PMID 37080222, Figure 2B |
| RATIONALE-306 | first line | 37080222 | TAP | 10% | TAP < 10% | 319 | 0.770 (0.590–1.010) | TAP subgroup reported descriptively; not included in multiplicity-adjusted analyses | TAP thresholds were derived from the same randomized cohort. TAP was analyzed separately from CPS and TC/TPS, and the single-trial contrast was reported descriptively. | PMID 37080222, Figure 2B |
| RATIONALE-306 | first line | 40075024 | TAP | 1% | TAP > =1%, final analysis | 481 | 0.660 (0.540–0.810) | TAP subgroup reported descriptively; not included in multiplicity-adjusted analyses | The post hoc TAP ≥ 1 analysis used the same randomized cohort; no complementary TAP < 1 estimate was reported. | PMID 40075024, Figure 2 |
| ATTRACTION-3 | second or later line | 31582355 | tumor proportion score / tumor-cell PD-L1 expression | 5% | <5% | 273 | 0.780 (0.600–1.030) | Secondary cutoff or category reported descriptively; not included in multiplicity-adjusted analyses | Multiple PD-L1 cutoffs from the same trial are correlated; each model used one prespecified cutoff or contrast per trial family. | PMID 31582355, Figure 3 (HR/CI and subgroup denominators); Online Supplement, Table S2 |
| ATTRACTION-3 | second or later line | 31582355 | tumor proportion score / tumor-cell PD-L1 expression | 5% | ≥5% | 146 | 0.730 (0.510–1.060) | Secondary cutoff or category reported descriptively; not included in multiplicity-adjusted analyses | Multiple PD-L1 cutoffs from the same trial are correlated; each model used one prespecified cutoff or contrast per trial family. | PMID 31582355, Figure 3 (HR/CI and subgroup denominators); Online Supplement, Table S2 |
| ATTRACTION-3 | second or later line | 31582355 | tumor proportion score / tumor-cell PD-L1 expression | 10% | <10% | 298 | 0.800 (0.620–1.040) | Secondary cutoff or category reported descriptively; not included in multiplicity-adjusted analyses | Multiple PD-L1 cutoffs from the same trial are correlated; each model used one prespecified cutoff or contrast per trial family. | PMID 31582355, Figure 3 (HR/CI and subgroup denominators); Online Supplement, Table S2 |
| ATTRACTION-3 | second or later line | 31582355 | tumor proportion score / tumor-cell PD-L1 expression | 10% | ≥10% | 121 | 0.690 (0.460–1.040) | Secondary cutoff or category reported descriptively; not included in multiplicity-adjusted analyses | Multiple PD-L1 cutoffs from the same trial are correlated; each model used one prespecified cutoff or contrast per trial family. | PMID 31582355, Figure 3 (HR/CI and subgroup denominators); Online Supplement, Table S2 |
| ATTRACTION-3 | second or later line | 35294546 | tumor proportion score / tumor-cell PD-L1 expression | 1% | <1% | 216 | 0.850 (0.630–1.140) | Included in a scoring-system- and cutoff-specific interaction analysis; one compatible estimate per trial family | The long-term update represented the same trial family; the ≥1%/<1% tumor-cell pair supplied the updated cutoff. | PMID 35294546, Supplementary Figure S1A |
| ATTRACTION-3 | second or later line | 35294546 | tumor proportion score / tumor-cell PD-L1 expression | 1% | ≥1% | 203 | 0.700 (0.520–0.950) | Included in a scoring-system- and cutoff-specific interaction analysis; one compatible estimate per trial family | The long-term update represented the same trial family; the ≥1%/<1% tumor-cell pair supplied the updated cutoff. | PMID 35294546, Supplementary Figure S1B |
| ESCORT | second or later line | 32416073 | tumor-cell PD-L1 expression / tumor proportion score | 1% | <1% | 247 | 0.820 (0.620–1.090) | Included in a scoring-system- and cutoff-specific interaction analysis; one compatible estimate per trial family | Multiple PD-L1 cutoffs from the same trial are correlated; each model used one prespecified cutoff or contrast per trial family. | PMID 32416073, Figure 2B (overall-survival subgroup analysis) |
| ESCORT | second or later line | 32416073 | tumor-cell PD-L1 expression / tumor proportion score | 1% | ≥1% | 191 | 0.580 (0.420–0.810) | Included in a scoring-system- and cutoff-specific interaction analysis; one compatible estimate per trial family | Multiple PD-L1 cutoffs from the same trial are correlated; each model used one prespecified cutoff or contrast per trial family. | PMID 32416073, Figure 2B (overall-survival subgroup analysis) |
| ESCORT | second or later line | 32416073 | tumor-cell PD-L1 expression / tumor proportion score | 5% | <5% | 355 | 0.740 (0.580–0.940) | Secondary cutoff or category reported descriptively; not included in multiplicity-adjusted analyses | Multiple PD-L1 cutoffs from the same trial are correlated; each model used one prespecified cutoff or contrast per trial family. | PMID 32416073, Figure 2B (overall-survival subgroup analysis) |
| ESCORT | second or later line | 32416073 | tumor-cell PD-L1 expression / tumor proportion score | 5% | ≥5% | 83 | 0.510 (0.300–0.840) | Secondary cutoff or category reported descriptively; not included in multiplicity-adjusted analyses | Multiple PD-L1 cutoffs from the same trial are correlated; each model used one prespecified cutoff or contrast per trial family. | PMID 32416073, Figure 2B (overall-survival subgroup analysis) |
| ESCORT | second or later line | 32416073 | tumor-cell PD-L1 expression / tumor proportion score | 10% | <10% | 377 | 0.710 (0.570–0.890) | Secondary cutoff or category reported descriptively; not included in multiplicity-adjusted analyses | Multiple PD-L1 cutoffs from the same trial are correlated; each model used one prespecified cutoff or contrast per trial family. | PMID 32416073, Figure 2B (overall-survival subgroup analysis) |
| ESCORT | second or later line | 32416073 | tumor-cell PD-L1 expression / tumor proportion score | 10% | ≥10% | 61 | 0.600 (0.320–1.080) | Secondary cutoff or category reported descriptively; not included in multiplicity-adjusted analyses | Multiple PD-L1 cutoffs from the same trial are correlated; each model used one prespecified cutoff or contrast per trial family. | PMID 32416073, Figure 2B (overall-survival subgroup analysis) |
| ORIENT-2 | second or later line | 35165274 | TPS | 1% | <1% | 87 | 0.793 (0.490–1.277) | Included in a scoring-system- and cutoff-specific interaction analysis; one compatible estimate per trial family | Multiple PD-L1 cutoffs from the same trial are correlated; each model used one prespecified cutoff or contrast per trial family. | PMID 35165274, Figure 3 (overall-survival subgroup analysis) |
| ORIENT-2 | second or later line | 35165274 | TPS | 1% | ≥1% | 54 | 0.876 (0.453–1.708) | Included in a scoring-system- and cutoff-specific interaction analysis; one compatible estimate per trial family | Multiple PD-L1 cutoffs from the same trial are correlated; each model used one prespecified cutoff or contrast per trial family. | PMID 35165274, Figure 3 (overall-survival subgroup analysis) |
| ORIENT-2 | second or later line | 35165274 | TPS | 10% | <10% | 113 | 0.763 (0.498–1.167) | Secondary cutoff or category reported descriptively; not included in multiplicity-adjusted analyses | Multiple PD-L1 cutoffs from the same trial are correlated; each model used one prespecified cutoff or contrast per trial family. | PMID 35165274, Figure 3 (overall-survival subgroup analysis) |
| ORIENT-2 | second or later line | 35165274 | TPS | 10% | ≥10% | 28 | 1.068 (0.413–2.760) | Secondary cutoff or category reported descriptively; not included in multiplicity-adjusted analyses | Multiple PD-L1 cutoffs from the same trial are correlated; each model used one prespecified cutoff or contrast per trial family. | PMID 35165274, Figure 3 (overall-survival subgroup analysis) |
| ORIENT-2 | second or later line | 35165274 | CPS | 1 | <1 | 28 | 1.303 (0.513–3.273) | Single-trial later-line CPS ≥ 1 vs <1 contrast; reported descriptively and not included in multiplicity adjustment | Multiple PD-L1 cutoffs from the same trial are correlated; each model used one prespecified cutoff or contrast per trial family. | PMID 35165274, Figure 3 (overall-survival subgroup analysis) |
| ORIENT-2 | second or later line | 35165274 | CPS | 1 | ≥1 | 113 | 0.729 (0.473–1.124) | Single-trial later-line CPS ≥ 1 vs <1 contrast; reported descriptively and not included in multiplicity adjustment | Multiple PD-L1 cutoffs from the same trial are correlated; each model used one prespecified cutoff or contrast per trial family. | PMID 35165274, Figure 3 (overall-survival subgroup analysis) |
| ORIENT-2 | second or later line | 35165274 | CPS | 10 | <10 | 81 | 0.950 (0.560–1.579) | Included in a scoring-system- and cutoff-specific interaction analysis; one compatible estimate per trial family | Multiple PD-L1 cutoffs from the same trial are correlated; each model used one prespecified cutoff or contrast per trial family. | PMID 35165274, Figure 3 (overall-survival subgroup analysis) |
| ORIENT-2 | second or later line | 35165274 | CPS | 10 | ≥10 | 60 | 0.738 (0.398–1.410) | Included in a scoring-system- and cutoff-specific interaction analysis; one compatible estimate per trial family | Multiple PD-L1 cutoffs from the same trial are correlated; each model used one prespecified cutoff or contrast per trial family. | PMID 35165274, Figure 3 (overall-survival subgroup analysis) |
| KEYNOTE-181 | second or later line | 33026938 | combined positive score (CPS) | 10 | ≥10 | 167 | 0.640 (0.460–0.900) | Included in a scoring-system- and cutoff-specific interaction analysis; one compatible estimate per trial family | Only PD-L1 estimates explicitly restricted to SCC/ESCC were eligible from this mixed-histology trial. | PMID 33026938, Figure 3B (squamous cell carcinoma subgroup) |
| KEYNOTE-181 | second or later line | 33026938 | combined positive score (CPS) | 10 | <10 | 228 | 0.880 (0.660–1.160) | Included in a scoring-system- and cutoff-specific interaction analysis; one compatible estimate per trial family | Only PD-L1 estimates explicitly restricted to SCC/ESCC were eligible from this mixed-histology trial. | PMID 33026938, Figure 3B (squamous cell carcinoma subgroup) |
| KEYNOTE-181 | second or later line | 34973513 | combined positive score (CPS) | 1 | PD-L1 CPS ≥ 1 | 280 | 0.570 (0.440–0.750) | Nested regional or extension analysis used only for sensitivity analyses; not included in multiplicity-adjusted analyses | The pooled Asian cohort included 231 participants from global KEYNOTE-181 and 109 from the China extension; it replaced the global KEYNOTE-181 contribution in the same model. | PMID 34973513, Figure 2 (Asian ESCC subgroup) |
| KEYNOTE-181 | second or later line | 34973513 | combined positive score (CPS) | 1 | PD-L1 CPS < 1 | 55 | 0.990 (0.560–1.720) | Nested regional or extension analysis used only for sensitivity analyses; not included in multiplicity-adjusted analyses | The pooled Asian cohort included 231 participants from global KEYNOTE-181 and 109 from the China extension; it replaced the global KEYNOTE-181 contribution in the same model. | PMID 34973513, Figure 2 (Asian ESCC subgroup) |

## Table S6 (continued). Extracted PD-L1 subgroup estimates and source locations

| **Trial** | **Line** | **PMID** | **Scoring system** | **Cutoff** | **Category** | **N** | **HR (95% CI)** | **Analysis role** | **Trial-family/overlap note** | **Exact source location** |
| --- | --- | --- | --- | --- | --- | --- | --- | --- | --- | --- |
| KEYNOTE-181 | second or later line | 34973513 | combined positive score (CPS) | 5 | PD-L1 CPS ≥ 5 | 212 | 0.560 (0.410–0.760) | Nested regional or extension analysis used only for sensitivity analyses; not included in multiplicity-adjusted analyses | The pooled Asian cohort included 231 participants from global KEYNOTE-181 and 109 from the China extension; it replaced the global KEYNOTE-181 contribution in the same model. | PMID 34973513, Figure 2 (Asian ESCC subgroup) |
| KEYNOTE-181 | second or later line | 34973513 | combined positive score (CPS) | 5 | PD-L1 CPS < 5 | 123 | 0.770 (0.520–1.130) | Nested regional or extension analysis used only for sensitivity analyses; not included in multiplicity-adjusted analyses | The pooled Asian cohort included 231 participants from global KEYNOTE-181 and 109 from the China extension; it replaced the global KEYNOTE-181 contribution in the same model. | PMID 34973513, Figure 2 (Asian ESCC subgroup) |
| RATIONALE-302 | second or later line | 35442766 | tumor area positivity (TAP) | 10% | TAP ≥ 10% | 157 | 0.540 (0.360–0.790) | TAP subgroup reported descriptively; not included in multiplicity-adjusted analyses | TAP categories were derived from the same randomized cohort. TAP was analyzed separately from CPS and TC/TPS, and the single-trial contrast was reported descriptively. | PMID 35442766, Figure 2B: OS in PD-L1 TAP ≥ 10% |
| RATIONALE-302 | second or later line | 35442766 | tumor area positivity (TAP) | 10% | TAP < 10% | 256 | 0.820 (0.620–1.090) | TAP subgroup reported descriptively; not included in multiplicity-adjusted analyses | TAP categories were derived from the same randomized cohort. TAP was analyzed separately from CPS and TC/TPS, and the single-trial contrast was reported descriptively. | PMID 35442766, Figure 2C: OS in PD-L1 TAP < 10% |
| RATIONALE-302 | second or later line | 35442766 | tumor area positivity (TAP) | not evaluable | TAP unknown | 99 | 0.670 (0.410–1.120) | TAP subgroup reported descriptively; not included in multiplicity-adjusted analyses | TAP categories were derived from the same randomized cohort. TAP was analyzed separately from CPS and TC/TPS, and the single-trial contrast was reported descriptively. | PMID 35442766, Figure 2D: OS in PD-L1 TAP unknown |

Multiple thresholds and reports from one trial are correlated observations from the same randomized family. CPS, tumor-cell/TPS, TAP, vCPS, and study-specific systems were not assumed interchangeable.

## Table S7. Post hoc interaction analyses and multiplicity adjustment

The post hoc analysis included 30 line-specific interaction hypotheses. Data were available for 28: 21 contrasts from at least two independent trial families were pooled and included in the Holm and Bonferroni adjustments, seven single-trial contrasts were reported descriptively, and two could not be evaluated. All adjusted P values for the 21 pooled interactions were 1.000. These hypotheses were not prespecified in the PROSPERO registration.

### Table S7A. Analysis status for 30 post hoc interaction hypotheses

| **ID** | **Line** | **Factor and contrast** | **PROSPERO prespecified** | **Eligible k** | **Status** | **Included in multiplicity adjustment** | **Reason / analytical role** |
| --- | --- | --- | --- | --- | --- | --- | --- |
| H01 | First line | Sex: Female vs male | No | 6 | Pooled | Yes | At least two independent all-comer trial families; pooled using REML with modified Hartung-Knapp inference and included in the Holm and Bonferroni adjustments. |
| H02 | First line | Age: ≥65 vs <65 years | No | 6 | Pooled | Yes | At least two independent all-comer trial families; pooled using REML with modified Hartung-Knapp inference and included in the Holm and Bonferroni adjustments. |
| H03 | First line | ECOG PS: ECOG 1 vs ECOG 0 | No | 6 | Pooled | Yes | At least two independent all-comer trial families; pooled using REML with modified Hartung-Knapp inference and included in the Holm and Bonferroni adjustments. |
| H04 | First line | Hepatic metastasis: Present vs absent | No | 3 | Pooled | Yes | At least two independent all-comer trial families; pooled using REML with modified Hartung-Knapp inference and included in the Holm and Bonferroni adjustments. |
| H05 | First line | Smoking status: Ever vs never smoker | No | 1 | Single-trial descriptive | No | One eligible trial family; reported descriptively and not included in multiplicity adjustment. |
| H06 | First line | Baseline body weight: ≥60 vs <60 kg | No | 2 | Pooled | Yes | At least two independent all-comer trial families; pooled using REML with modified Hartung-Knapp inference and included in the Holm and Bonferroni adjustments. |
| H07 | First line | Trial-reported race: White vs Asian/other, as reported | No | 1 | Single-trial descriptive | No | One eligible trial family; reported descriptively and not included in multiplicity adjustment. |
| H08 | First line | Disease extent: Metastatic vs locally advanced | No | 4 | Pooled | Yes | At least two independent all-comer trial families; pooled using REML with modified Hartung-Knapp inference and included in the Holm and Bonferroni adjustments. |
| H09 | First line | Geographic region: Outside Asia vs Asia | No | 2 | Pooled | Yes | At least two independent all-comer trial families; pooled using REML with modified Hartung-Knapp inference and included in the Holm and Bonferroni adjustments. |
| H10 | First line | Prior radiotherapy/definitive local therapy: Yes vs no, as defined in each trial | No | 3 | Pooled | Yes | At least two independent all-comer trial families; pooled using REML with modified Hartung-Knapp inference and included in the Holm and Bonferroni adjustments. |
| H11 | First line | Number of metastatic organs: ≥2 vs ≤1 organs | No | 2 | Pooled | Yes | At least two independent all-comer trial families; pooled using REML with modified Hartung-Knapp inference and included in the Holm and Bonferroni adjustments. |
| H12 | Second or later line | Sex: Female vs male | No | 5 | Pooled | Yes | At least two independent all-comer trial families; pooled using REML with modified Hartung-Knapp inference and included in the Holm and Bonferroni adjustments. |
| H13 | Second or later line | Age: ≥65 vs <65 years | No | 4 | Pooled | Yes | At least two independent all-comer trial families; pooled using REML with modified Hartung-Knapp inference and included in the Holm and Bonferroni adjustments. |
| H14 | Second or later line | ECOG PS: ECOG 1 vs ECOG 0 | No | 5 | Pooled | Yes | At least two independent all-comer trial families; pooled using REML with modified Hartung-Knapp inference and included in the Holm and Bonferroni adjustments. |
| H15 | Second or later line | Hepatic metastasis: Present vs absent | No | 2 | Pooled | Yes | At least two independent all-comer trial families; pooled using REML with modified Hartung-Knapp inference and included in the Holm and Bonferroni adjustments. |
| H16 | Second or later line | Smoking status: Ever vs never smoker | No | 1 | Single-trial descriptive | No | One eligible trial family; reported descriptively and not included in multiplicity adjustment. |
| H17 | Second or later line | Baseline body weight: ≥60 vs <60 kg | No | 0 | Unavailable | No | No valid complementary all-comer subgroup pair in this treatment-line domain. |
| H18 | Second or later line | Trial-reported race: White vs Asian/other, as reported | No | 2 | Pooled | Yes | At least two independent all-comer trial families; pooled using REML with modified Hartung-Knapp inference and included in the Holm and Bonferroni adjustments. |
| H19 | Second or later line | Disease extent: Metastatic vs locally advanced | No | 0 | Unavailable | No | No valid complementary all-comer subgroup pair in this treatment-line domain. |
| H20 | Second or later line | Geographic region: Outside Asia vs Asia | No | 2 | Pooled | Yes | At least two independent all-comer trial families; pooled using REML with modified Hartung-Knapp inference and included in the Holm and Bonferroni adjustments. |
| H21 | Second or later line | Prior radiotherapy/definitive local therapy: Yes vs no, as defined in each trial | No | 1 | Single-trial descriptive | No | One eligible trial family; reported descriptively and not included in multiplicity adjustment. |
| H22 | Second or later line | Number of metastatic organs: ≥2 vs ≤1 organs | No | 2 | Pooled | Yes | At least two independent all-comer trial families; pooled using REML with modified Hartung-Knapp inference and included in the Holm and Bonferroni adjustments. |
| H23 | First line | PD-L1 CPS: CPS ≥ 1 vs <1 | No | 4 | Pooled | Yes | At least two independent all-comer trial families; pooled using REML with modified Hartung-Knapp inference and included in the Holm and Bonferroni adjustments. |
| H24 | First line | PD-L1 CPS: CPS ≥ 10 vs <10 | No | 4 | Pooled | Yes | At least two independent all-comer trial families; pooled using REML with modified Hartung-Knapp inference and included in the Holm and Bonferroni adjustments. |
| H25 | First line | PD-L1 TC/TPS: Tumor-cell PD-L1/TPS: ≥1% vs <1% | No | 4 | Pooled | Yes | At least two independent all-comer trial families; pooled using REML with modified Hartung-Knapp inference and included in the Holm and Bonferroni adjustments. |
| H26 | Second or later line | PD-L1 CPS: CPS ≥ 1 vs <1 | No | 1 | Single-trial descriptive | No | One eligible trial family (ORIENT-2); RHR derived from complementary subgroup HRs and reported descriptively without meta-analysis or multiplicity adjustment. |
| H27 | Second or later line | PD-L1 CPS: CPS ≥ 10 vs <10 | No | 2 | Pooled | Yes | At least two independent all-comer trial families; pooled using REML with modified Hartung-Knapp inference and included in the Holm and Bonferroni adjustments. |
| H28 | Second or later line | PD-L1 TC/TPS: Tumor-cell PD-L1/TPS: ≥1% vs <1% | No | 3 | Pooled | Yes | At least two independent all-comer trial families; pooled using REML with modified Hartung-Knapp inference and included in the Holm and Bonferroni adjustments. |
| H29 | First line | PD-L1 TAP: TAP > =10% vs <10% | No | 1 | Single-trial descriptive | No | One eligible trial family; the TAP contrast was reported descriptively and analyzed separately from CPS and TC/TPS. |
| H30 | Second or later line | PD-L1 TAP: TAP > =10% vs <10% | No | 1 | Single-trial descriptive | No | One eligible trial family; the TAP contrast was reported descriptively and analyzed separately from CPS and TC/TPS. |

### Table S7B. Results of 21 pooled interaction analyses with multiplicity adjustment

| **ID** | **Line** | **Factor and contrast** | **k** | **RHR (95% CI)** | **Raw P** | **Holm P** | **Bonferroni P** | **τ²** | **I²** |
| --- | --- | --- | --- | --- | --- | --- | --- | --- | --- |
| H01 | First line | Sex: Female vs male | 6 | 1.125 (0.786–1.611) | 0.436 | 1.000 | 1.000 | 0.0050 | 4.2% |
| H02 | First line | Age: ≥65 vs <65 years | 6 | 0.914 (0.727–1.150) | 0.360 | 1.000 | 1.000 | 0.0000 | 0.0% |
| H03 | First line | ECOG PS: ECOG 1 vs ECOG 0 | 6 | 1.057 (0.816–1.370) | 0.605 | 1.000 | 1.000 | 0.0000 | 0.0% |
| H04 | First line | Hepatic metastasis: Present vs absent | 3 | 0.826 (0.434–1.570) | 0.328 | 1.000 | 1.000 | 0.0000 | 0.0% |
| H06 | First line | Baseline body weight: ≥60 vs <60 kg | 2 | 0.826 (0.104–6.574) | 0.450 | 1.000 | 1.000 | 0.0000 | 0.0% |
| H08 | First line | Disease extent: Metastatic vs locally advanced | 4 | 1.013 (0.504–2.034) | 0.958 | 1.000 | 1.000 | 0.0949 | 49.9% |
| H09 | First line | Geographic region: Outside Asia vs Asia | 2 | 0.993 (0.148–6.644) | 0.971 | 1.000 | 1.000 | 0.0000 | 0.0% |
| H10 | First line | Prior radiotherapy/definitive local therapy: Yes vs no, as defined in each trial | 3 | 1.099 (0.554–2.178) | 0.614 | 1.000 | 1.000 | 0.0000 | 0.0% |
| H11 | First line | Number of metastatic organs: ≥2 vs ≤1 organs | 2 | 0.800 (0.053–12.109) | 0.486 | 1.000 | 1.000 | 0.0462 | 50.1% |
| H12 | Second or later line | Sex: Female vs male | 5 | 0.808 (0.523–1.250) | 0.246 | 1.000 | 1.000 | 0.0000 | 0.0% |
| H13 | Second or later line | Age: ≥65 vs <65 years | 4 | 0.969 (0.679–1.383) | 0.797 | 1.000 | 1.000 | 0.0000 | 0.0% |
| H14 | Second or later line | ECOG PS: ECOG 1 vs ECOG 0 | 5 | 0.882 (0.579–1.343) | 0.453 | 1.000 | 1.000 | 0.0450 | 39.9% |
| H15 | Second or later line | Hepatic metastasis: Present vs absent | 2 | 0.909 (0.065–12.731) | 0.725 | 1.000 | 1.000 | 0.0000 | 0.0% |
| H18 | Second or later line | Trial-reported race: White vs Asian/other, as reported | 2 | 0.726 (0.030–17.561) | 0.422 | 1.000 | 1.000 | 0.0000 | 0.0% |
| H20 | Second or later line | Geographic region: Outside Asia vs Asia | 2 | 1.069 (0.015–76.675) | 0.875 | 1.000 | 1.000 | 0.1699 | 75.0% |
| H22 | Second or later line | Number of metastatic organs: ≥2 vs ≤1 organs | 2 | 0.841 (0.110–6.402) | 0.474 | 1.000 | 1.000 | 0.0000 | 0.0% |
| H23 | First line | PD-L1 CPS: CPS ≥ 1 vs <1 | 4 | 0.671 (0.399–1.128) | 0.092 | 1.000 | 1.000 | 0.0000 | 0.0% |
| H24 | First line | PD-L1 CPS: CPS ≥ 10 vs <10 | 4 | 0.859 (0.598–1.235) | 0.276 | 1.000 | 1.000 | 0.0098 | 18.8% |
| H25 | First line | PD-L1 TC/TPS: Tumor-cell PD-L1/TPS: ≥1% vs <1% | 4 | 0.835 (0.448–1.557) | 0.425 | 1.000 | 1.000 | 0.0953 | 63.4% |
| H27 | Second or later line | PD-L1 CPS: CPS ≥ 10 vs <10 | 2 | 0.738 (0.060–9.031) | 0.366 | 1.000 | 1.000 | 0.0000 | 0.0% |
| H28 | Second or later line | PD-L1 TC/TPS: Tumor-cell PD-L1/TPS: ≥1% vs <1% | 3 | 0.799 (0.429–1.490) | 0.262 | 1.000 | 1.000 | 0.0000 | 0.0% |

## **Table S8. Departures from the registered protocol and post hoc analyses**

| **Item** | **Registration status** | **Description** |
| --- | --- | --- |
| Registration identifier | CRD42024551725; first submitted 28 May 2024; registered 8 June 2024 | The registered record lists searches through 5 June 2024. Registration occurred 3 days after that search date, although the first submission preceded it; the review is not described as prospectively registered relative to searching. |
| Final database search | 1 August 2026 | PubMed, Embase, the Cochrane Central Register of Controlled Trials, and Web of Science were searched through this date. |
| Original review framework | Registered | Eligibility criteria and the overall-survival outcome were retained. |
| Report linkage by trial family | Not prespecified | Reports were grouped by randomized cohort to prevent duplicate inclusion of participants across 31 reports from 14 trial families. |
| Treatment-line stratification | Not prespecified | First-line combination and later-line monotherapy were analyzed separately; the cross-line synthesis was descriptive. |
| Random-effects method | Not prespecified | REML with modified Hartung-Knapp inference was applied uniformly, with q* = max(1, q); the model was not selected according to observed heterogeneity. |
| Within-trial RHR | Not prespecified | Treatment-by-subgroup interactions were estimated from within-trial ratios of hazard ratios. |
| Multiplicity adjustment | Not prespecified | Holm and Bonferroni adjustments were applied to 21 pooled interactions; single-trial contrasts were reported descriptively. |
| PD-L1 subgroup framework | Not prespecified | PD-L1 scoring systems and cutoffs were analyzed separately by treatment line and data cutoff. |
| ASTRUM-007 | Not prespecified | The trial was excluded from primary all-comer OS and clinical-interaction analyses and included only in CPS ≥ 1-enriched sensitivity analyses. |
| Certainty of evidence | Not assessed | Certainty of evidence was not assessed. |
| Clinical and PD-L1 interaction hypotheses | Not prespecified | Thirty line-specific hypotheses were defined for the post hoc analysis; 21 pooled interactions were included in multiplicity adjustment. All interaction analyses were exploratory. |

## **Table S9.** RoB 2 domain-level judgments and supporting rationale for overall survival

| **Trial family** | **D1** | **D2** | **D3** | **D4** | **D5** | **Overall** | **Rationale and evidence sources** |
| --- | --- | --- | --- | --- | --- | --- | --- |
| CheckMate 648 | Low | Some concerns | Low | Low | Low | Some concerns | D1 (Low): Central web-based interactive response technology used concealed, stratified block randomization; the three randomized groups were broadly balanced at baseline. D2 (Some concerns): The trial was open label and treatment initiation was uneven (310/321, 322/325, and 304/324 treated). D3 (Low): OS was analyzed in the all-randomized population, with surviving participants censored at the last confirmed alive date; no material arm-specific loss of vital-status information was identified. D4 (Low): OS was time from randomization to death. D5 (Low): The protocol/SAP material specifies the primary populations, testing hierarchy, alpha allocation, interim analyses, and Cox/log-rank methods; the reported all-population OS result follows that plan. Evidence sources: PMID 35108470, main report and online supplementary appendix, protocol, and statistical analysis plan. |
| KEYNOTE-590 | Low | Low | Low | Low | Low | Low | D1 (Low): Central interactive randomization with concealed block allocation and stratification was used; baseline features were balanced. D2 (Low): Placebo masking minimized trial-context deviations, and all 749 randomized participants were retained in the ITT OS analysis despite small numbers not receiving treatment. D3 (Low): All randomized participants entered the OS analysis and censoring rules were specified; no important differential missing vital status was evident. D4 (Low): OS was death from any cause and was measured uniformly under blinded, placebo-controlled conduct. D5 (Low): The SAP specified the ESCC and all-participant OS populations, interim/final timing, multiplicity, and analytical methods before reporting. Evidence sources: PMID 34454674, main report. |
| ESCORT-1st | Low | Low | Low | Low | Low | Low | D1 (Low): Central IWRS block randomization with stratification and concealed assignment was used; baseline characteristics were balanced. D2 (Low): Double-blind placebo control minimized deviations, and the primary efficacy analysis included all 596 randomized participants as assigned. D3 (Low): All randomized participants were analyzed, with only one reported loss to follow-up and a prespecified last-known-alive censoring rule. D4 (Low): OS was measured from randomization to death and is objective; identical ascertainment applied in the masked groups. D5 (Low): The published report and online supplementary material include the original and final protocols, amendments, and statistical analysis plan; the primary OS analysis and multiplicity plan were prespecified. Evidence sources: PMID 34519801, main report and online supplementary appendix, protocol, and statistical analysis plan. |
| GEMSTONE-304 | Low | Low | Low | Low | Low | Low | D1 (Low): An interactive response system implemented stratified 2:1 randomization; baseline characteristics were generally balanced. D2 (Low): Double-blind placebo control was maintained; all randomized participants remained in the ITT analysis, including one participant who received chemotherapy without the investigational antibody. D3 (Low): The ITT analysis included all 540 randomized participants, with only one reported loss to follow-up and explicit survival censoring. D4 (Low): OS was time from randomization to death and was measured uniformly under double-blind conduct. D5 (Low): The report describes protocol changes made before analysis and a prespecified SAP with interim timing and multiplicity control; the OS result follows the named plan. Evidence sources: PMID 38302715, main report. |
| SKYSCRAPER-08 | Low | Low | Low | Low | Low | Low | D1 (Low): Central IVRS permuted-block randomization with prespecified stratification and concealed allocation was used; randomized groups were balanced. D2 (Low): Double-blind placebo treatment was maintained through final OS for participants and investigators, and OS was analyzed in all randomized participants. D3 (Low): Losses and withdrawals were small and balanced, and all randomized participants were included in the OS analysis with prespecified censoring. D4 (Low): OS was objectively defined from randomization to death and assessed uniformly under masking. D5 (Low): The protocol/SAP and prespecified OS analysis, sensitivity analyses, and testing plan are described; no evidence of outcome/model selection was identified. Evidence sources: PMID 41449142, main report and online supplementary material. |
| ASTRUM-007 | Low | High | Low | Low | Low | High | D1 (Low): Central IWRS randomization with stratification and masked placebo control was used; no baseline pattern suggested a randomization problem. D2 (High): 15 of 183 placebo-assigned participants received serplulimab because of a study-drug distribution error. This was a documented trial-context deviation, was confined to the control group, exposed 8.2% of that group to active checkpoint inhibition, and was likely to dilute the OS contrast. D3 (Low): All randomized participants were retained in ITT; survival was censored at the last known alive date, with no material differential loss identified. D4 (Low): OS was death from any cause with common ascertainment; the objective endpoint is not readily influenced by treatment knowledge. D5 (Low): The protocol/SAP specified OS, ITT analysis, interim/final timing, and alpha control; the reported result follows those methods. Evidence sources: PMID 36732627, main report. |
| JUPITER-06 | Low | Low | Low | Low | Low | Low | D1 (Low): An independent statistician generated variable blocks implemented via IWRS, with stratification and concealment; baseline characteristics were balanced. D2 (Low): Double-blind identical placebo treatment was used, crossover was not permitted, all randomized participants received protocol therapy, and ITT analysis preserved assignment. D3 (Low): All 514 randomized participants were analyzed, with only one reported loss to follow-up in the control arm. D4 (Low): OS was time from randomization to death from any cause and was ascertained identically under masking. D5 (Low): The prespecified interim OS analysis, multiplicity control, and ITT methods are detailed; the reported result follows the named plan. Evidence sources: PMID 35245446, main report. |
| ORIENT-15 | Low | Low | Low | Low | Low | Low | D1 (Low): Central IWRS randomization with stratification and concealed masked assignment was used; baseline groups were balanced. D2 (Low): Patients, investigators, and sponsor were blinded, and efficacy was analyzed in all 659 randomized participants as assigned. D3 (Low): All randomized participants were included in the OS analysis with a common survival follow-up and censoring approach; no material differential missing vital status was identified. D4 (Low): OS was time from randomization to death and was measured identically under masked conduct. D5 (Low): For the all-randomized OS result judged here, the endpoint and analysis were prespecified and unchanged. Evidence sources: PMID 35440464, main report. |
| RATIONALE-306 | Low | Low | Low | Low | Low | Low | D1 (Low): Interactive response technology implemented stratified permuted-block randomization with concealed assignment; baseline groups were balanced. D2 (Low): Patients, investigators, and sponsor were masked; only two participants per group did not receive treatment and all randomized participants remained in ITT. D3 (Low): All randomized participants were included in the OS analysis and no important differential missing survival information was identified. D4 (Low): OS was based on death and measured uniformly under double-blind conduct. D5 (Low): The protocol/SAP, prespecified interim timing, O'Brien-Fleming boundary, testing hierarchy, and ITT analysis are reported; no selective result choice was identified. Evidence sources: PMID 37080222, main report and online supplementary material. |
| ATTRACTION-3 | Low | Low | Low | Low | Low | Low | D1 (Low): A vendor-generated block sequence was implemented through IWRS with stratification and concealment; baseline groups were balanced. D2 (Low): The study was open label, but only one participant in each arm did not receive treatment, and all 419 randomized participants were analyzed as assigned. D3 (Low): All randomized participants were included in OS analysis with a prespecified last-known-alive censoring rule; no important differential missingness was evident. D4 (Low): OS was death from any cause. D5 (Low): The published report and online supplementary material include protocol and statistical-analysis-plan versions and amendments. Evidence sources: PMID 31582355, main report and online supplementary appendix, protocol, and statistical analysis plan. |
| ESCORT | Low | Some concerns | Low | Low | Low | Some concerns | D1 (Low): Central response-technology block randomization with stratification and concealed allocation was described; baseline groups did not suggest a randomization failure. D2 (Some concerns): The open-label trial's primary OS analysis excluded randomized participants who did not start treatment: 448/457 were analyzed, with exclusions strongly unbalanced (1/229 camrelizumab versus 8/228 chemotherapy). D3 (Low): Nine of 457 randomized participants (2.0%) were absent from the reported FAS. D4 (Low): OS was death from any cause and is objective; open-label knowledge is unlikely to alter measurement of death. D5 (Low): OS was the stated primary endpoint and the FAS/Cox/log-rank analysis was explicitly described; the report indicates protocol availability and no competing OS result was identified. Evidence sources: PMID 32416073, main report. |
| ORIENT-2 | Low | Some concerns | Low | Low | Some concerns | Some concerns | D1 (Low): IWRS with mixed block sizes and ECOG stratification was used, providing concealed randomized assignment; no baseline evidence suggested failure. D2 (Some concerns): The trial was open label and treatment initiation was differential (94/95 versus 87/95). D3 (Low): All randomized participants entered the ITT OS analysis, including those who refused assigned treatment; surviving participants were censored at last follow-up. D4 (Low): OS was time from randomization to death. D5 (Some concerns): OS was the stated primary endpoint and the analysis is described, but the published report and available online supplement did not include a full protocol or dated statistical analysis plan to verify that the exact analysis plan was finalized before unblinded outcome data in this open-label phase 2 trial. Evidence sources: PMID 35165274, main report and available online supplementary material. |
| KEYNOTE-181 | Some concerns | Some concerns | Low | Low | Low | Some concerns | D1 (Some concerns): The report states randomized, histology- and region-stratified assignment and shows balanced groups, but the published report and available online supplement do not provide the sequence-generation or allocation-concealment mechanism in sufficient detail. D2 (Some concerns): The trial was open label and treatment non-initiation was markedly differential (314/314 received pembrolizumab versus 296/314 receiving chemotherapy). D3 (Low): All 628 randomized participants were included in OS analysis. D4 (Low): OS was death from any cause and is objective. D5 (Low): SCC OS was one of three explicitly prospectively specified primary populations, with an SAP-defined interim/final testing and multiplicity strategy. Evidence sources: PMID 33026938, main report and available online supplementary material. |
| RATIONALE-302 | Low | Some concerns | Low | Low | Low | Some concerns | D1 (Low): Central IRT randomization with stratification and concealed assignment was used. D2 (Some concerns): The trial was open label and treatment non-initiation was markedly differential (255/256 received tislelizumab versus 240/256 receiving chemotherapy). D3 (Low): All 512 randomized participants formed the ITT OS analysis; no material differential missing vital status was identified. D4 (Low): OS was death from any cause. D5 (Low): The published report and online supplementary material include the protocol and full statistical analysis plan; the SAP states it was finalized before unmasking and specifies primary OS, ITT population, censoring, and analyses. Evidence sources: PMID 35442766, main report and online supplementary appendix, protocol, and statistical analysis plan. |

Judgments apply to overall survival at the randomized trial-family level. Linked updates, corrections, biomarker reports, and regional reports informed the judgments without creating additional trial rows. Sources are identified by PMID and publicly available report component. Two reviewers (B.W. and X.H.) independently completed all domain-level and overall judgments. Disagreements were resolved by consensus, with unresolved judgments adjudicated by Z.S. D1–D5 definitions are provided in the Figure 2 caption.

## Table S10. Heterogeneity and prediction-interval statistics for primary pooled HR and RHR models

Prediction intervals are shown only for models with at least five independent trial families. When τ² = 0, a reported prediction interval can coincide with the corresponding confidence interval. Q, Cochran's heterogeneity statistic; RHR, ratio of hazard ratios.

| **Model / domain** | **Effect** | **k** | **Estimate (95% CI)** | **τ²** | **I²** | **Q** | **Q P** | **95% prediction interval** |
| --- | --- | --- | --- | --- | --- | --- | --- | --- |
| First-line single ICI + chemotherapy: Overall survival; ICI-containing regimen vs control | HR | 7 | 0.702 (0.640–0.771) | 0.0000 | 0.0% | 2.779 | 0.836 | 0.640-0.771 |
| Second- or later-line single-agent ICI: Overall survival; ICI-containing regimen vs control | HR | 5 | 0.737 (0.641–0.848) | 0.0000 | 0.0% | 1.063 | 0.900 | 0.641-0.848 |
| First-line interaction: Sex; Female vs male | RHR | 6 | 1.125 (0.786–1.611) | 0.0050 | 4.2% | 4.669 | 0.458 | 0.753-1.682 |
| First-line interaction: Age; ≥65 vs <65 years | RHR | 6 | 0.914 (0.727–1.150) | 0.0000 | 0.0% | 2.747 | 0.739 | 0.727-1.150 |
| First-line interaction: ECOG PS; ECOG 1 vs ECOG 0 | RHR | 6 | 1.057 (0.816–1.370) | 0.0000 | 0.0% | 1.339 | 0.931 | 0.816-1.370 |
| First-line interaction: Hepatic metastasis; Present vs absent | RHR | 3 | 0.826 (0.434–1.570) | 0.0000 | 0.0% | 0.452 | 0.798 | Not reported (k < 5) |
| First-line interaction: Baseline body weight; ≥60 vs <60 kg | RHR | 2 | 0.826 (0.104–6.574) | 0.0000 | 0.0% | 0.162 | 0.687 | Not reported (k < 5) |
| First-line interaction: Disease extent; Metastatic vs locally advanced | RHR | 4 | 1.013 (0.504–2.034) | 0.0949 | 49.9% | 5.967 | 0.113 | Not reported (k < 5) |
| First-line interaction: Geographic region; Outside Asia vs Asia | RHR | 2 | 0.993 (0.148–6.644) | 0.0000 | 0.0% | 0.002 | 0.960 | Not reported (k < 5) |
| First-line interaction: Prior radiotherapy/definitive local therapy; Yes vs no, as defined in each trial | RHR | 3 | 1.099 (0.554–2.178) | 0.0000 | 0.0% | 0.854 | 0.653 | Not reported (k < 5) |
| First-line interaction: Number of metastatic organs; ≥2 vs ≤1 organs | RHR | 2 | 0.800 (0.053–12.109) | 0.0462 | 50.1% | 2.005 | 0.157 | Not reported (k < 5) |
| Later-line interaction: Sex; Female vs male | RHR | 5 | 0.808 (0.523–1.250) | 0.0000 | 0.0% | 2.245 | 0.691 | 0.523-1.250 |
| Later-line interaction: Age; ≥65 vs <65 years | RHR | 4 | 0.969 (0.679–1.383) | 0.0000 | 0.0% | 2.760 | 0.430 | Not reported (k < 5) |
| Later-line interaction: ECOG PS; ECOG 1 vs ECOG 0 | RHR | 5 | 0.882 (0.579–1.343) | 0.0450 | 39.9% | 6.329 | 0.176 | 0.428-1.819 |
| Later-line interaction: Hepatic metastasis; Present vs absent | RHR | 2 | 0.909 (0.065–12.731) | 0.0000 | 0.0% | 0.491 | 0.483 | Not reported (k < 5) |
| Later-line interaction: Trial-reported race; White vs Asian/other, as reported | RHR | 2 | 0.726 (0.030–17.561) | 0.0000 | 0.0% | 0.015 | 0.902 | Not reported (k < 5) |
| Later-line interaction: Geographic region; Outside Asia vs Asia | RHR | 2 | 1.069 (0.015–76.675) | 0.1699 | 75.0% | 4.002 | 0.045 | Not reported (k < 5) |
| Later-line interaction: Number of metastatic organs; ≥2 vs ≤1 organs | RHR | 2 | 0.841 (0.110–6.402) | 0.0000 | 0.0% | 0.334 | 0.564 | Not reported (k < 5) |
| First-line interaction: PD-L1 CPS; CPS ≥ 1 vs <1 | RHR | 4 | 0.671 (0.399–1.128) | 0.0000 | 0.0% | 1.543 | 0.672 | Not reported (k < 5) |
| First-line interaction: PD-L1 CPS; CPS ≥ 10 vs <10 | RHR | 4 | 0.859 (0.598–1.235) | 0.0098 | 18.8% | 3.606 | 0.307 | Not reported (k < 5) |
| First-line interaction: PD-L1 TC/TPS; Tumor-cell PD-L1/TPS: ≥1% vs <1% | RHR | 4 | 0.835 (0.448–1.557) | 0.0953 | 63.4% | 8.620 | 0.035 | Not reported (k < 5) |
| Later-line interaction: PD-L1 CPS; CPS ≥ 10 vs <10 | RHR | 2 | 0.738 (0.060–9.031) | 0.0000 | 0.0% | 0.019 | 0.889 | Not reported (k < 5) |
| Later-line interaction: PD-L1 TC/TPS; Tumor-cell PD-L1/TPS: ≥1% vs <1% | RHR | 3 | 0.799 (0.429–1.490) | 0.0000 | 0.0% | 0.926 | 0.629 | Not reported (k < 5) |

# Supplementary figures

**Conventions for Supplementary Figures S1-S19. Unless otherwise stated, HRs and RHRs are shown with 95% CIs. RHRs are calculated as the HR in the first-named category divided by the HR in the second-named category; values below 1 indicate greater relative benefit from the ICI-containing regimen in the first-named category. Pooled estimates use REML random-effects models with modified Hartung-Knapp inference; the primary RHR variance assumes zero within-trial covariance. First-line and second- or later-line models are displayed separately. CI, confidence interval; CPS, combined positive score; FL, first line; HR, hazard ratio; ICI, immune checkpoint inhibitor; LL, later line; mKH, modified Hartung-Knapp; PD-L1, programmed death ligand 1; REML, restricted maximum likelihood; RHR, ratio of hazard ratios; TAP, tumor area positivity; TC/TPS, tumor-cell expression/tumor proportion score.**

**Figure S1. Sex subgroup-specific HRs and within-trial interaction estimates for overall survival. RHR numerator/denominator: female/male. ASTRUM-007 contributes only to the CPS ≥ 1-conditional sensitivity shown here.**

**Figure S2. Age subgroup-specific HRs and within-trial interaction estimates for overall survival. RHR numerator/denominator: ≥65 years/<65 years. ASTRUM-007 contributes only to the CPS ≥ 1-conditional sensitivity shown here.**

**Figure S3. ECOG performance-status subgroup-specific HRs and within-trial interaction estimates for overall survival. RHR numerator/denominator: ECOG 1/ECOG 0. ASTRUM-007 contributes only to the CPS ≥ 1-conditional sensitivity shown here.**

**Figure S4. Hepatic-metastasis subgroup-specific HRs and within-trial interaction estimates for overall survival. RHR numerator/denominator: present/absent. ASTRUM-007 contributes only to the CPS ≥ 1-conditional sensitivity shown here.**

**Figure S5. Smoking-status subgroup-specific HRs and within-trial interaction estimates for overall survival. RHR numerator/denominator: ever smoker/never smoker. ASTRUM-007 contributes only to the CPS ≥ 1-conditional sensitivity shown here.**

**Figure S6.** Baseline-body-weight subgroup-specific HRs and within-trial interaction estimates for overall survival. RHR numerator/denominator: ≥60 kg/<60 kg. ATTRACTION-3 reported no numerical body-weight/BMI subgroup HR; no value was imputed.

**Figure S7.** Trial-reported-race subgroup-specific HRs and within-trial interaction estimates for overall survival. RHR numerator/denominator: White/Asian or other.

**Figure S8. Disease-extent subgroup-specific HRs and within-trial interaction estimates for overall survival. RHR numerator/denominator: metastatic/locally advanced. ASTRUM-007 contributes only to the CPS ≥ 1-conditional sensitivity shown here.**

**Figure S9.** Geographic-region subgroup-specific HRs and within-trial interaction estimates for overall survival. RHR numerator/denominator: outside Asia/Asia.

**Figure S10.** Prior-radiotherapy/definitive-therapy subgroup-specific HRs and within-trial interaction estimates for overall survival. RHR numerator/denominator: yes/no.

**Figure S11.** Metastatic-burden subgroup-specific HRs and within-trial interaction estimates for overall survival. RHR numerator/denominator: ≥2 sites or organs/≤1 site or organ.

**Figure S12. PD-L1 interaction estimates by scoring system, cutoff, treatment line, and data cutoff. Single-trial contrasts are shown descriptively and were not pooled.**

**Figure S13. Secondary PD-L1 cutoff interaction analyses. Alternative thresholds and enriched or report-specific definitions were analyzed separately; the cross-line TAP display is descriptive.**

**Figure S14. Availability of PD-L1 scoring-system and cutoff definitions across trial families. Blue, eligible complementary pair; amber, one or non-complementary eligible estimate; gray, no eligible estimate.**

**Figure S15. Extended overall-survival sensitivity analyses. The display includes the primary treatment-line pools, the CPS ≥ 1-enriched ASTRUM-007 analysis, the descriptive cross-line synthesis, and separate dual-checkpoint strategies. Rows are analyzed separately and do not represent randomized comparisons across treatment lines or mechanisms.**

**Figure S16. Sensitivity of selected interaction estimates to alternative meta-analytic methods. Disease-extent and metastatic-burden models use all-comer inputs; the CPS ≥ 1 model includes four trials with compatible definitions.**

**Figure S17. Sensitivity of selected interaction estimates to assumed within-trial correlations (ρ = -0.25, 0, and +0.25). The hepatic-metastasis model excludes ASTRUM-007; PD-L1 models include estimates with compatible scoring systems and cutoffs.**

**Figure S18. Availability of eligible clinical subgroup estimates by trial family. Blue, strict complementary primary all-comer pair; amber, reported estimate without a strict pair; gray, not reported or not eligible; purple, ASTRUM-007 CPS ≥ 1-conditional sensitivity estimate.**

**Figure S19. Leave-one-out sensitivity analysis of the first-line CPS ≥ 1 versus < 1 interaction estimate.**
